# An innate pathogen sensing strategy involving ubiquitination of bacterial surface proteins

**DOI:** 10.1126/sciadv.ade1851

**Published:** 2023-03-22

**Authors:** Shruti Apte, Smita Bhutda, Sourav Ghosh, Kuldeep Sharma, Thomas E. Barton, Soham Dibyachintan, Osheen Sahay, Suvapriya Roy, Akash Raj Sinha, Harikrishna Adicherla, Jyotirmoy Rakshit, Shiying Tang, Akshay Datey, Shweta Santra, Jincy Joseph, Sreeja Sasidharan, Sven Hammerschmidt, Dipshikha Chakravortty, Marco R. Oggioni, Manas Kumar Santra, Daniel R. Neill, Anirban Banerjee

**Affiliations:** ^1^ Department of Biosciences and Bioengineering, Indian Institute of Technology Bombay, Mumbai 400076, Maharashtra, India.; ^2^ Department of Clinical Infection, Microbiology and Immunology, University of Liverpool, L69 7BE Liverpool, UK.; ^3^ Cancer Biology and Epigenetics Laboratory, National Centre for Cell Science, Ganeshkhind Road, Pune 411007, Maharashtra, India.; ^4^ CSIR-Centre for Cellular and Molecular Biology, Uppal Road, Habsiguda, Hyderabad 500007 Telangana, India.; ^5^ Department of Genetics and Genome Biology, University of Leicester, Leicester, UK.; ^6^ Department of Microbiology and Cell Biology, Indian Institute of Science, Bengaluru 560012, Karnataka, India.; ^7^ Department of Molecular Genetics and Infection Biology, Interfaculty Institute of Genetics and Functional Genomics, Center for Functional Genomics of Microbes, University of Greifswald, D-17487 Greifswald, Germany.

## Abstract

Sensing of pathogens by ubiquitination is a critical arm of cellular immunity. However, universal ubiquitination targets on microbes remain unidentified. Here, using in vitro, ex vivo, and in vivo studies, we identify the first protein-based ubiquitination substrates on phylogenetically diverse bacteria by unveiling a strategy that uses recognition of degron-like motifs. Such motifs form a new class of intra-cytosolic pathogen-associated molecular patterns (PAMPs). Their incorporation enabled recognition of nonubiquitin targets by host ubiquitin ligases. We find that SCF^FBW7^ E3 ligase, supported by the regulatory kinase, glycogen synthase kinase 3β, is crucial for effective pathogen detection and clearance. This provides a mechanistic explanation for enhanced risk of infections in patients with chronic lymphocytic leukemia bearing mutations in F-box and WD repeat domain containing 7 protein. We conclude that exploitation of this generic pathogen sensing strategy allows conservation of host resources and boosts antimicrobial immunity.

## Teaser

Degron mediated ubiquitination of bacterial surface proteins fosters sensing and clearance of diverse pathogens.

## INTRODUCTION

Pathogenic invasion triggers a battery of immune responses stimulated by surveillance mechanisms of the host. This is generally initiated via recognition of conserved microbial molecular structures known as pathogen-associated molecular patterns (PAMPs). Effective sensing of these PAMPs by pattern recognition receptors (PRRs) rapidly induces a variety of host immune responses via the activation of complex signaling pathways triggering pathogen clearance. To date, several classes of PRRs, such as Toll-like receptors, Retinoic acid-inducible gene I (RIG-I)–like receptors, NOD-like receptors, and DNA receptors (cytosolic sensors for DNA), have been discovered and characterized (*1*). These PRRs are at the forefront of both extracellular and intracellular pathogen recognition and sense various classes of molecules in microbes including proteins, lipids, carbohydrates, and nucleic acids (*2*). This is pivotal to halt disease progression and promote host survival.

Surveillance of the intracellular milieu for restriction of pathogen proliferation is critical to preserve cytosolic sterility. A breach of such defense mechanisms not only provides the pathogen a refuge from extracellular innate immunity but also offers an opportunity for rapid multiplication and dissemination within the host (*3*). Potent pathogen sensing mechanisms and cell-autonomous defense systems are therefore critical to restrict invasive pathogens. Ubiquitination is one strategy that plays a pivotal role in pathogen recognition and elimination (*4*). The degradative pathway dictated by ubiquitination acts as a final frontier against cytosol dwelling bacteria that often evade the classical endocytic killing by rupturing pathogen-containing vacuoles to invade host cytosol. Several host E3 ubiquitin ligases have been identified to decorate cargos including intracellular pathogens with poly-ubiquitin (Ub) chains (*5*), and although few bacterial targets such as outer membrane proteins have been detected (*6*), extensive knowledge regarding substrate identification strategy remains limited. A recent study has demonstrated secreted effector proteins to contain ubiquitin-associated domain (UBA) in *Mycobacterium tuberculosis* (Mtb) that passively recruit ubiquitin moieties, ultimately delivering pathogen to Microtubule-associated protein 1A/1B-light chain 3 (LC3) -associated autophagosomes (*7*). Alongside, unusual ubiquitin substrates like lipopolysaccharide (LPS) and glycan have been elegantly illustrated in couple of bacterial pathogens, showcasing the versatility of ubiquitination substrates (*8*, *9*). Complementarily, *Rickettsia parkeri* was found to actively modify surface proteins, protecting it from ubiquitination and subsequent killing (*10*). Together, these independent studies emphasize the significance of the surface localization of the ubiquitin substrate. However, the identity of a proteinaceous substrate in pathogen and how they could be precisely identified by host E3 ligase remain elusive.

Quite a few host ubiquitin ligases, such as leucine-rich repeat and sterile α-motif containing 1 (LRSAM1), Parkin, Ring finger protein 166 (RNF166), RNF213, Ariadne RING-BetweenRING-RING (RBR) E3-ubiquitin protein ligase 1 (ARIH1), SMAD-specific E3-ubiquitin protein ligase 1 (Smurf1), and Skip-Cullin-F-box protein 2 containing complex (SCF^FBXO2^) are reported to decorate pathogen or pathogen-containing vacuoles with a variety of ubiquitin chain topologies (*8*, *9*, *11*–*18*). Notably, in particular, LRSAM1 through auto-ubiquitination possibly generates a robust ubiquitin signal around the bacteria to recruit the autophagic machinery (*19*, *20*). The ubiquitin ligases responsible for pathogen marking are also involved in maintaining cellular homeostasis, creating an extremely frugal system for efficient and optimal resource utilization. Among various ubiquitin chain topologies formed on the pathogen, M1-Ub decoration primarily drives induction of inflammation (*21*), while both K48- and K63-Ub chain topologies effectively target microbes toward the autophagy or proteasomal system, respectively (*22*). We have recently demonstrated that K48-Ub chain has more dominant antibacterial effect compared to K63-Ub (*23*). In general, cellular proteins destined for proteasomal degradation are tagged by K48-Ub chain–specific ligases. The key signal for substrate recognition by such ligases is principally directed by a degron motif (*24*).

In this study, we identify the existence of degron motifs in surface proteins of phylogenetically diverse bacteria of both Gram-positive and Gram-negative origin. The targeting of such substrates by ubiquitination machinery propels efficient pathogen elimination from the host cell. Using this, we demonstrate the conversion of a nonubiquitinable surface protein into an ubiquitin substrate by engineering degron insertion to promote bacterial clearance. This simple yet generic principle for identifying bacterial substrate potentially serves as a conserved mechanism of cytosolic pathogen recognition, promising to be efficient and multipurpose in fending off bacterial infections.

## RESULTS

### K48-Ub chain promotes sensing of cytosolic bacterial pathogens

**Fig. 1.**
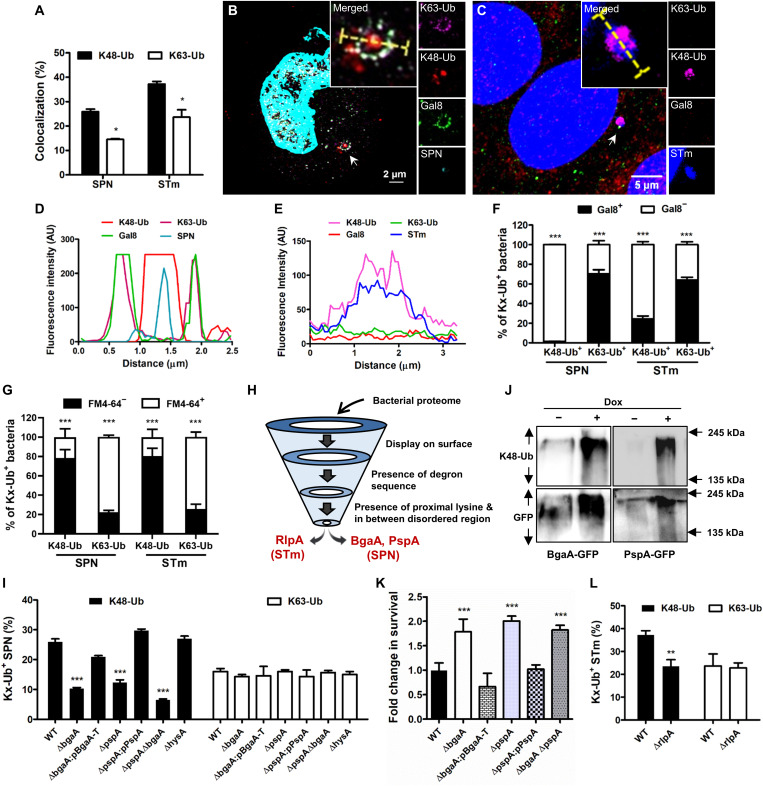
Identification of bacterial surface proteins as substrates for ubiquitination. (**A**) Association of SPN and STm with K48-Ub or K63-Ub. *n* > 100 bacteria per coverslip. (**B**) SIM image showing association of SPN (cyan) with K48-Ub (red) inside a damaged endosome marked with Gal8 (green) and K63-Ub (magenta). Scale bar, 2 μm. (**C**) Confocal micrograph showing decoration of cytosolic STm (blue) with K48-Ub (magenta), devoid of K63-Ub (red) or Gal8 (green). Arrowheads in (B) and (C) depict event shown in inset. Scale bar, 5 μm. (**D** and **E**) Fluorescent line scans across the yellow dashed line in the merged inset in (B) and (C). (**F**) Percentage of cytosolic (Gal8^−^) or damaged vacuole-bound (Gal8^+^) SPN or STm marked with K48 and K63-Ub. *n* > 50 bacteria per coverslip. (**G**) Percentage of cytosolic (FM4-64^−^) or membrane-associated (FM4-64^+^) SPN and STm marked with K48 or K63-Ub. *n* > 50 bacteria per coverslip. (**H**) Schematic of screening for putative K48-Ub target proteins in bacteria. (**I**) Comparison of association of K48 or K63-Ub with different SPN strains. *n* > 100 bacteria per coverslip. (**J**) Immunoblot demonstrating ex vivo K48 ubiquitination of BgaA-T and PspA. (**K**) Fold change in intracellular survival of different mutant strains normalized to WT SPN. (**L**) Association of K48-Ub with STm. *n* > 100 bacteria per coverslip. For all experiments mentioned above, A549 and HeLa cells are infected with SPN and STm for 9 and 3 hours, respectively. Statistical significance was assessed by two-way analysis of variance (ANOVA; Bonferroni test) (A, F, and G) and one-way ANOVA (Dunnett’s test) (H and K) while comparing K48-Ub versus K63-Ub for (A), Gal8^−^ versus Gal8^+^ for (F), FM4-64^+^ versus FM4-64^−^ for (G), and mutant strains versus WT for (I) and (K) and two-tailed unpaired Student’s *t* test (L). *P < 0.05; **P < 0.01; ***P < 0.005. Data are means ± SD of *N* = 3 independent biological replicates. AU, arbitrary units; GFP, green fluorescent protein.

Upon sensing cytosolic invasion by pathogens, the host marks them with poly-Ub chains to trigger their clearance (*22*). Since such poly-Ub chains are primarily composed of K48- and K63-Ub, we first explored the predominance and spatial location of these chain types on two phylogenetically distinct pathogens, *Streptococcus pneumoniae* (SPN) and *Salmonella enterica* serovar Typhimurium (STm), which cause pneumonia and gastroenteritis in humans, respectively. For these pathogens, survival and proliferation within the host cell cytosol have been documented (*13*, *25*). Using ubiquitin linkage–specific antibodies, we observed that a significantly higher proportion of intracellular bacteria were marked with K48-Ub chain type (~26% for SPN and 37% for STm) in contrast to K63-Ub (Fig. 1A). Analysis of spatial location by structured illumination microscopy (SIM) indicated that cytosolic (free of vacuolar remnants) or cytosol-exposed bacteria (within damaged endosome) are primarily associated with K48-Ub, while K63-Ub signal was located on damaged endosomes, marked with Galectin-8 (Gal8; endosome damage sensing marker) (Fig. 1, B to E) (*26*). About 99 and ~76% of K48-ubiquitinated SPN and STm, respectively, were devoid of Gal8 (Fig. 1F). The bacterial presence in the cytosol was further validated by transmission electron microscopy (TEM) and immunostaining with a membrane marker FM4-64 (fig. S1, A to F). We found 78.4 and 80.4% K48-Ub–positive SPN and STm, respectively, devoid of any membrane association, while 77.7 and 74.4% K63-Ub–positive SPN and STm, respectively, were confined within vacuole (Fig. 1G). Collectively, these findings suggested that coating of bacterial surface with K48-Ub chains is a major pathogen sensing mechanism used by the host for recognizing cytosol-dwelling microbes.

### Degron is a generic code for bacterial ubiquitination

We next attempted to identify the substrate for K48 ubiquitination on the bacterial surface. Critically, host E3 ubiquitin ligases, reported to be involved in bacterial ubiquitination, are also implicated in crucial cellular functions (*12*, *14*) where K48-Ub chains act as a major signal for cellular proteostasis. We hypothesized that similar principles could be adopted by the host for identification of K48-Ub substrate on the bacterial surface. For host proteins, the presence of a tripartite motif (a primary degron sequence followed by a proximal lysine residue and a disordered region in-between) is reported to be a prerequisite for K48 ubiquitination (*24*). We screened surface proteins of SPN for the presence of similar features (Fig. 1H), identifying BgaA and PspA as putative targets for ubiquitination (Fig. 1H and fig. S2, A and B). BgaA is a β-galactosidase reported to function as an adhesin for SPN, while PspA is a choline-binding protein that binds lactoferrin and is required for complement evasion (*27*, *28*). We observed ~50 to 53% reduction in association of K48-Ub chain type for both Δ*bgaA* and Δ*pspA* mutants, without any change in K63-Ub levels (Fig. 1I). This reduction was pronounced (~75%) in a double-knockout strain (Δ*bgaA*Δ*pspA*), suggesting the nonredundant nature of these ubiquitin substrates (Fig. 1I). Moreover, expression of BgaA-T (a truncated version of the protein, consisting of amino acids 1 to 1049) and PspA in host cells leads to their ubiquitination with K48-Ub topology (Fig. 1J). The validity of the predicted targets was confirmed by complementation, and the model was strengthened by using Δ*hysA* (SPN surface protein that does not fulfill tripartite degron criteria) mutant as a control to score K48-Ub association levels (Fig. 1I).

We next explored the effect of K48-Ub decoration on bacterial clearance. The absence of K48-Ub substrates impeded bacterial clearance, resulting in significantly improved intracellular persistence for both mutant SPN strains (~1.8-fold for Δ*bgaA* and ~2-fold for Δ*pspA*) (Fig. 1K). The universality of our substrate prediction approach was validated by the identification of several surface-exposed proteins in various other pathogens as putative substrates for ubiquitination (table S1). One such putative candidate, an outer membrane protein RlpA on STm was confirmed as a target for the host ubiquitination machinery, as the Δ*rlpA* mutant exhibited ~1.5-fold reduced association with K48-Ub compared to wild-type (WT) STm (Fig. 1L). This finding established the broad applicability of our substrate selection strategy. To the best of our knowledge, these are the first bacterial surface proteins reported to be recognized by host ubiquitination machinery for pathogen sensing and clearance.

### A tripartite motif is prerequisite for precise ubiquitin tagging of bacterial surface proteins

**Fig. 2.**
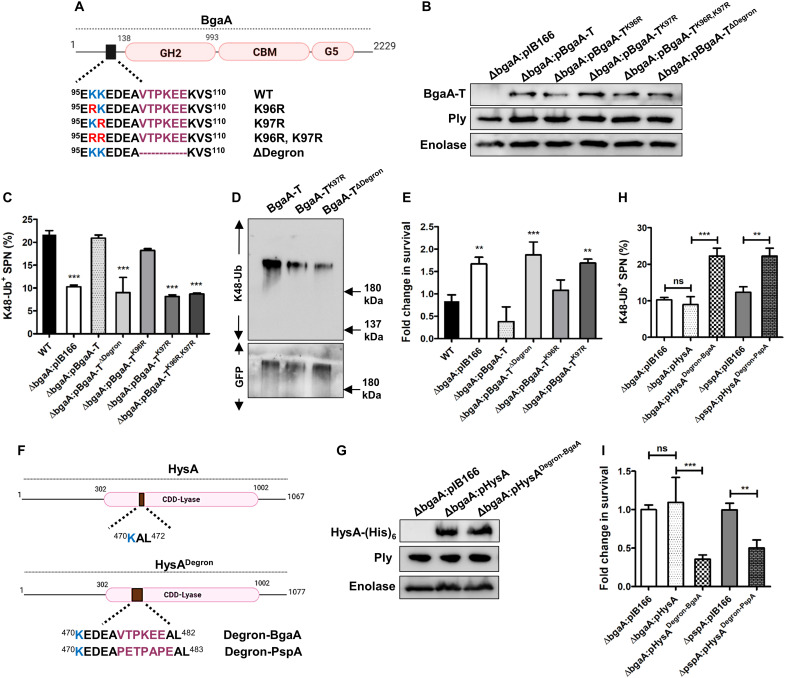
Significance of degron motif and proximal lysine in ubiquitination of BgaA. (**A**) Schematic diagram of BgaA depicting the presence of degron and proximal lysine residues. Also shown are different mutant BgaA variants used in the study. (**B**) Immunoblot showing expression of BgaA variants in Δ*bgaA* strains. Similar levels of pneumolysin (Ply) across strains ensure equal damage to endosome membrane. Enolase was used as a loading control. (**C**) Percentage of association of K48-Ub chain with different SPN strains at 9 hours after infection in A549 cells. Δ*bgaA*:pIB166 designates vector control. *n* > 100 bacteria per coverslip. (**D**) Ex vivo ubiquitination of BgaA-T, BgaA-T^K97R^, and BgaA-T^ΔDegron^. (**E**) Fold change in intracellular survival of different SPN strains in A549s normalized to WT at 9 hours after infection. (**F**) Schematic diagram of a non–K48-Ub target protein HysA and its variants following incorporation of degron sequence of BgaA and PspA. (**G**) Western blot showing expression of HysA and its Degron added variants (HysA^Degron-BgaA^ and HysA^Degron-PspA^) in Δ*bgaA* and Δ*pspA* backgrounds. Expression of Ply was similar across the strains, and enolase was used as loading control. (**H**) Percentage of association of K48-Ub with different SPN strains carrying variants of HysA at 9 hours after infection in A549 cells. *n* > 100 bacteria per coverslip. (**I**) Fold change in intracellular persistence ability of different SPN strains harboring HysA variants in A549s normalized to Δ*bgaA* or Δ*pspA* at 9 hours after infection. Statistical significance was assessed by one-way ANOVA followed by Dunnett’s test (C and E) or Tukey’s test (H and I). ns, nonsignificant; **P < 0.01; ***P < 0.005, versus WT. Data are means ± SD of *N* = 3 independent biological replicates.

Following substrate identification, we aimed to test the critical features of the tripartite motif which formed the backbone of our screen (Fig. 2A and fig. S2A). Deletion of the degron sequence (^102^VTPKEE^107^) in BgaA-T resulted in a ~50% drop in association of K48-Ub chain type, compared to WT SPN regardless of similar growth kinetics and cell adherence ability (Fig. 2C and fig. S3, A and B). This reduction was comparable to the Δ*bgaA* knockout strain, confirming the degron-specific phenotype. Apart from the degron sequence, the presence of a lysine residue in close vicinity is crucial for attachment of the ubiquitin moiety to the substrate. Since in BgaA, the degron sequence is accompanied by two proximal lysine residues (K96 and K97), we mutated both lysine residues (K96R and K97R), which resulted in 62% reduction in K48 ubiquitination compared to WT SPN (Fig. 2C). Individually, the K97R substitution led to a ~68% decrease in K48 ubiquitination, while K96R substitution resulted in only ~31% reduction (Fig. 2C). This result suggests that host E3 ligases show preference for a particular lysine residue for tagging ubiquitin substrates. Deletion of the degron sequence and mutation of the marked lysine residue (K97) inhibited SPN clearance, leading to a ~1.5- to 2-fold increase in bacterial persistence within airway epithelial cells (Fig. 2E). These observations were verified by expression of BgaA-T^K97R^ and BgaA-T^ΔDegron^ variants in host cells, where they exhibited considerably reduced K48 ubiquitination compared to BgaA-T (Fig. 2D). Notably, possibility of severe conformational change in BgaA-T^ΔDegron^ protein to affect ubiquitination was nullified by in silico prediction and circular dichroism (CD) spectroscopy of purified BgaA-T^ΔDegron^ protein, which showed similar structural signatures to BgaA-T (fig. S3, C and D). Like BgaA, degron sequence (^327^PETPAPE^333^) deletion and lysine mutation (K315R) in PspA also led to significantly reduced K48-Ub association, coupled with prolonged surviving ability (fig. S4, A to D).

We next engineered the SPN surface protein HysA, originally lacking a primary degron sequence, by addition of a degron sequence within a structurally disordered region of the protein that contain a lysine residue (Fig. 2, F and G, and fig. S2, C to E). This modification conferred recognition and K48 ubiquitination of the previously nonubiquitinable HysA protein. K48-Ub association levels of SPN strains carrying the engineered HysA protein (Δ*bgaA*:pHysA^Degron-BgaA^ and Δ*pspA*:pHysA^Degron-PspA^) were 2.2- and 3.2-fold higher compared to either Δ*bgaA* and Δ*bgaA*:pHysA or Δ*pspA* strains, respectively (Fig. 2H). The enhanced decoration of Δ*bgaA*:pHysA^Degron-BgaA^ and Δ*pspA*:pHysA^Degron-PspA^ strains with K48-Ub robbed SPN of the survival gain provided by the absence of degron in Δ*bgaA* and Δ*bgaA*:pHysA or Δ*pspA* (Fig. 2I). All the engineered SPN strains produced similar levels of the pore-forming toxin pneumolysin (Ply) (Fig. 2, B and G), which is a prerequisite for endomembrane damage and subsequent ubiquitination (*25*). This nullifies the possible contribution of low or extensive membrane damage promoting marked change of ubiquitination levels in mutant SPN strains. Collectively, these suggest that artificial addition of degron sequence promotes ubiquitin-mediated detection and elimination of pathogens. Notably, the degron sequence in BgaA was highly conserved across different pneumococcal serotypes (fig. S5A). However, in serotype 19F which is often associated with increased risk of death from bacteremic pneumonia and sepsis (*29*–*31*), the primary degron was found to be mutated (P104Q). We observed that mimicking this mutation in BgaA (fig. S5B) imparted poor ubiquitination and improved survival ability to Δ*bgaA*:pBgaA-T^P104Q^ compared to Δ*bgaA*:pBgaA-T (fig. S5, C and D). This highlights degron recognition as a strategy used by the host to protect itself against severe bacterial infections.

### SCF^FBW7^ is an antimicrobial E3 ubiquitin ligase

**Fig. 3.**
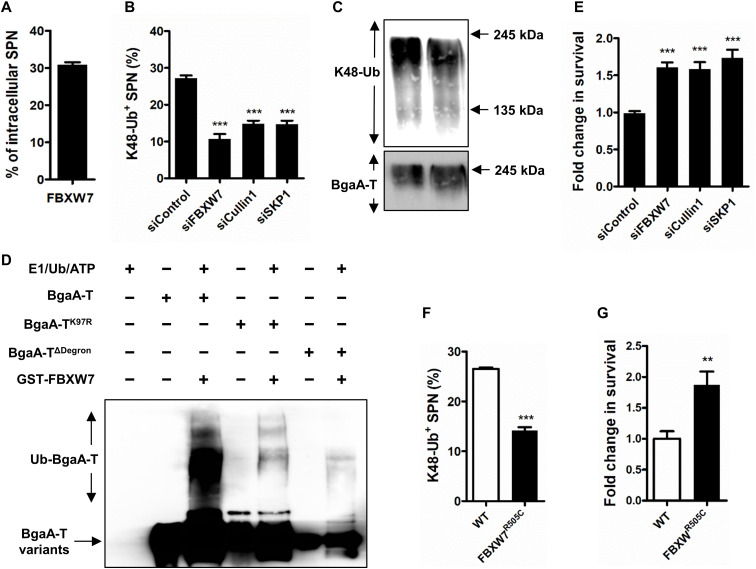
SCF^FBW7^ ubiquitinates surface proteins of cytosolic SPN. (**A**) Percentage of association of SPN with FBXW7 in A549 cells at 9 hours after infection. *n* > 100 bacteria per coverslip. (**B**) Percentage of decoration of SPN with K48-Ub following knockdown of FBXW7, Cullin1, and SKP1 in comparison to scrambled siRNA (siControl) transfected cells. *n* > 100 bacteria per coverslip. (**C**) Immunoblot showing ex vivo ubiquitination of BgaA-T in siCullin1-, siFBXW7-, and siSKP1-treated cells. (**D**) In vitro ubiquitination reaction demonstrating ubiquitination of BgaA-T, BgaA-T^K97R^, and BgaA-T^ΔDegron^. Recombinant active components of SCF complex were incubated with purified BgaA-T-His_6_ variants, E1, E2 (cdc34), Rbx1, ubiquitin, ATP, and FBXW7. Anti-BgaA and anti-GFP antibodies were used for immunoblotting. (**E**) Fold change in intracellular survival of SPN in A549 cells transfected with siFBXW7, siCullin1, and siSKP1 and normalized to siControl. (**F**) Percent association of SPN with K48-Ub in FBXW7^R505C^-mutated A549 cells. *n* > 100 bacteria per coverslip. (**G**) Fold change in intracellular survival efficiency of SPN in FBXW7^R505C^-expressing A549 cells compared to WT cells. Statistical significance was assessed by one-way ANOVA followed by Dunnett’s test (B and E) and two-tailed unpaired Student’s *t* test (F and G**)**. **P < 0.01; ***P < 0.005. Data are means ± SD of *N* = 3 independent biological replicates.

The canonical degron sequence present in the selected ubiquitin substrates is predicted to be identified by the SCF^FBW7^ E3 ubiquitin ligase complex (*24*), which is involved in regulation of cell cycle and growth (*32*). It is composed of two conserved proteins, S-phase kinase associated protein 1 (SKP1) and a member of the Cullin protein family, along with a variable F-box protein that provides substrate specificity (*33*). To verify involvement of SCF^FBW7^ in SPN ubiquitination, we first assessed association of FBXW7 with SPN. We found ~31% of intracellular SPN to be associated with FBXW7 upon immunofluorescence analysis (Fig. 3A and fig. S6A). Expectedly, FBXW7-positive SPN also colocalized with K48 ubiquitin (fig. S6B). To prove the involvement of SCF^FBW7^, labeling of the bacteria with K48-Ub chains was examined by immunofluorescence, following down-regulation of the expression of Cullin1, SKP1, and FBXW7 genes using targeted small interfering RNAs (siRNAs; fig. S7, A to C). In particular, FBXW7 silencing was validated by the cyclin E1 accumulation level in siFBXW7-treated cells (fig. S7F). We observed ~45 to 60% reduction in SPN association with K48-Ub in Cullin1, SKP1, and FBXW7 knockdown cells (Fig. 3B), which, in turn, led to ~1.6- to 1.75-fold increase in SPN persistence within host cells (Fig. 3E). The specific targeting of degron motif by SCF^FBW7^ was proved by unaltered differences in K48-Ub colocalization and survival ability of Δ*pspA*Δ*bgaA* and Δ*bgaA:*pBgaA-T^ΔDegron^ strains in siFBXW7-treated cells (fig. S8, A to D). These findings were substantiated by notable reductions in K48 ubiquitination of BgaA-T in host cells following knockdown of FBXW7 (Fig. 3C). Further, in vitro ubiquitination with purified BgaA-T (fig. S9, A to D) and the SCF complex components unambiguously demonstrates SCF^FBW7^ as the bona fide E3 ligase responsible for ubiquitination of BgaA. Recombinant SCF^FBW7^ was capable of ubiquitinating purified BgaA-T but failed to ubiquitinate the degron-deleted variant BgaA-T^ΔDegron^ or the lysine-to-arginine substitution variant BgaA-T^K97R^ (Fig. 3D). Moreover, host cells expressing FBXW7^R505C^ variant, which exhibits impaired recognition ability for cyclin E1 (a substrate of FBXW7) (fig. S7E), showed reduced (~50%) K48 ubiquitination of SPN, as well as ~2-fold higher survival of SPN compared to WT cells (Fig. 3, F and G). These experiments prove the key role of the SCF^FBW7^ E3 ligase in detection of cytosol dwelling pathogens and targeting them toward killing pathways.

### GSK3β-mediated phosphorylation of degron motif potentiates antimicrobial activity of SCF^FBW7^

**Fig. 4.**
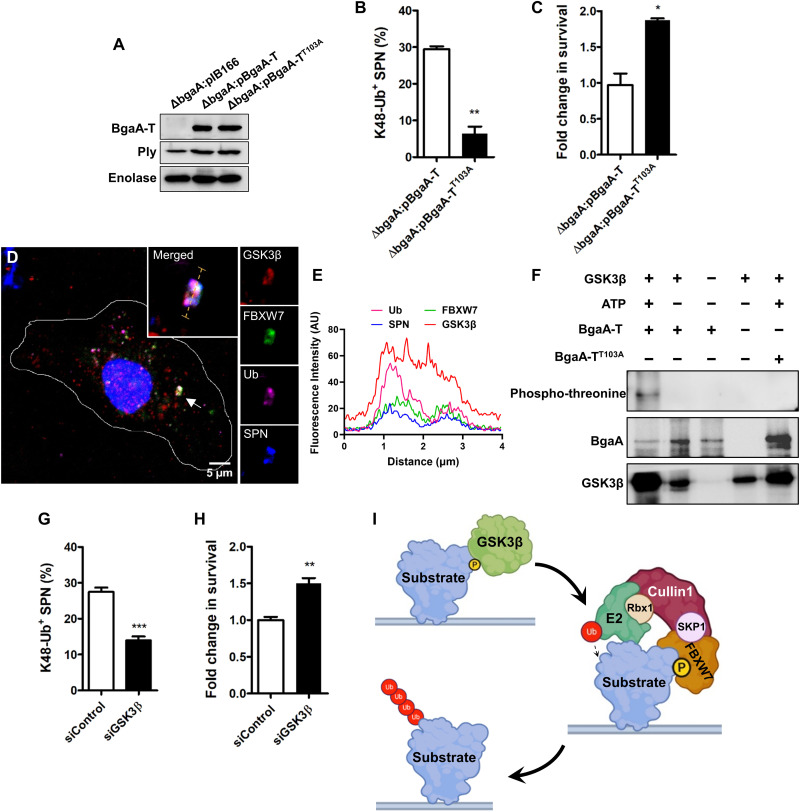
GSK3β-mediated phosphorylation is key for ubiquitination of SPN surface proteins. (**A**) Immunoblot analysis of BgaA complemented phosphodegron mutant variants. (**B**) Percent association of phosphodegron mutant SPN strain (Δ*bgaA*:pBgaA-T^T103A^) with K48-Ub in comparison to WT SPN in A549 cells at 9 hours after infection. *n* > 100 bacteria per coverslip. (**C**) Fold change in intracellular persistence ability of phosphodegron mutant SPN in A549s normalized to WT at 9 hours after infection. (**D**) Representative confocal micrograph demonstrating association of ubiquitinated SPN with FBXW7 and GSK3β. Scale bar, 5 μm. (**E**) Fluorescent line scan across the yellow dashed line in the merged inset in (D). (**F**) In vitro kinase assay assessing phosphorylation of BgaA-T and BgaA-T^T103A^ by recombinant GSK3β. Phosphorylated proteins were detected with anti-phospho threonine antibody. (**G**) Percentage of association of SPN with K48-Ub following knockdown of GSK3β. Cells transfected with scrambled siRNA (siControl) served as a control. *n* > 100 bacteria per coverslip. (**H**) Fold change in intracellular survival of SPN in A549 cells transfected with siGSK3β and normalized to siControl. (**I)** Schematic depicting phosphorylation of degron motif in bacterial surface protein by GSK3β, followed by recruitment and ubiquitination by SCF^FBW7^ E3 ligase. Scheme was designed with help of BioRender software. Statistical significance was assessed by two-tailed unpaired Student’s *t* test (B, C, G, and H). *P < 0.05; **P < 0.01. Data are means ± SD of *N* = 3 independent biological replicates.

In general, F-box proteins recognize phosphorylated substrate to promote their ubiquitination (*34*). We therefore investigated the likelihood and impact of phosphorylation of bacterial substrates on K48-Ub coating of the pathogen. Bioinformatics analysis revealed the presence of a putative phosphorylable threonine residue (^102^VT*PKEE^107^) within the degron sequence in BgaA. We observed that SPN strain harboring a BgaA-T^T103A^ mutation (Δ*bgaA*:pBgaA-T^T103A^) (Fig. 4A) manifested 71% reduced colocalization with K48-Ub compared to WT (Fig. 4B), revealing the relevance of phosphorylation in substrate recognition by the SCF complex. Critically, the decreased propensity of BgaA phosphorylation in Δ*bgaA*:pBgaA-T^T103A^ abrogated the host’s ability to eliminate intracellular bacterial loads (~1.8-fold) (Fig. 4C). In parallel to BgaA, a PspA degron variant (Δ*pspA*:pPspA^T329A^) also showed a 51% drop in K48-Ub colocalization that was associated with prolonged intracellular persistence (fig. S10, A to C). In general, SCF^FBW7^ target substrates have a threonine/serine (T/S*) next to a proline residue, which is phosphorylated by a proline-directed protein kinase, GSK3β (*35*–*37*). We therefore attempted to unravel the involvement of GSK3β in augmenting the substrate recognition. We first demonstrated that GSK3β is closely affiliated with ubiquitinated SPN, marked with FBXW7 (Fig. 4, D and E). Subsequently, by performing an in vitro kinase assay, we observed that GSK3β could phosphorylate recombinant BgaA-T, while the BgaA-T^T103A^ variant remained nonphosphorylated (Fig. 4F). This validated the identity of the threonine residue within the degron sequence of BgaA-T as a target for GSK3β-mediated phosphorylation. Targeted knockdown of GSK3β by siRNA (fig. S7D) led to ~58% reduction in K48 ubiquitination of SPN (Fig. 4G). This reduced ubiquitination, following down-regulation of GSK3β expression, resulted in diminished ability of the host to clear cell-invaded pathogens (~1.5-fold) (Fig. 4H) but did not show any effect on Δ*bgaA*:pBgaA-T^T103A^ (fig. S8, E and F). Collectively, this provides the first evidence of a host kinase, specifically GSK3β, regulating ubiquitination of bacterial surface proteins for efficient clearance of pathogens (Fig. 4I).

Ubiquitination of cytosolic pathogens imparts distinct fates for their elimination. In particular, K48 ubiquitination promotes targeting of substrates toward proteasomes (*22*). In similar lines, our results suggest association of ubiquitinated SPN with proteasomal subunit, β7 (fig. S11, A and C). Moreover, proteasomal inhibition by MG132 treatment improves persistence of WT SPN but does not alter the survival ability of Δ*pspA*Δ*bgaA*. Similar phenotypes were observed in case of STm and Δ*rlpA* mutant (fig. S11, B and D).

### Pathogen surveillance guided by degron protects the host from sepsis

**Fig. 5.**
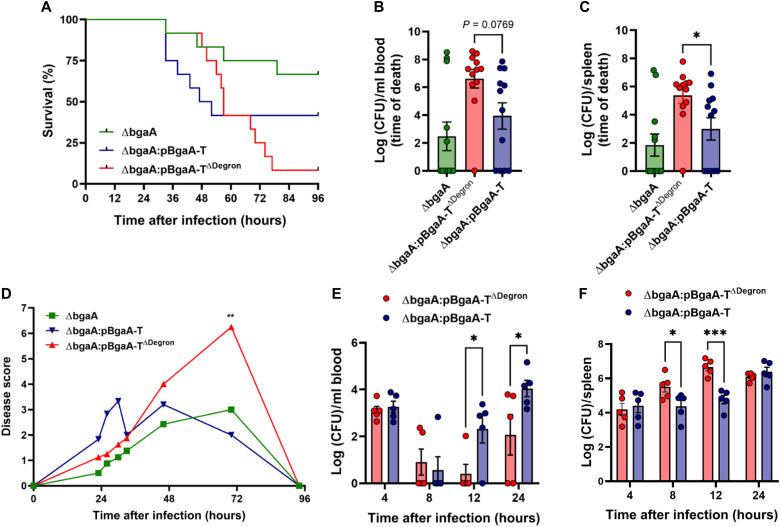
Recognition of the BgaA degron sequence aids control of SPN during sepsis. Mice were infected intravenously with 1 × 10^6^ colony-forming units (CFU) of different SPN strains in 100 μl of phosphate-buffered saline (PBS). (**A**) Survival following intravenous infection. *N* = 12 mice per group. (**B** and **C**) Blood (B) and spleen (C) CFU at time of death. (**D**) Physical signs of disease in infected mice. Scoring system takes into account physical appearance, weight, breathing rate, and natural and provoked behavior. (**E** and **F**) Bacterial burdens in blood (E) and spleen (F) over the first 24 hours after intravenous infection. In (E) and (F), only mice with detectable bacteria in blood or spleen at each time point were included. Statistical analysis was by one-way (B and C) or two-way (D to F) ANOVA, with correction for multiple comparisons. *P < 0.05: **P < 0.01; ***P < 0.001.

We then sought to determine the impact of SPN recognition via the cellular ubiquitination machinery on outcomes of infection. Using an established model of SPN sepsis (*38*), we compared the virulence of the Δ*bgaA* mutant with that of the WT SPN as well as strains complemented with either BgaA-T (Δ*bgaA*:pBgaA-T) or a version lacking the degron sequence (Δ*bgaA*:pBgaA-T^ΔDegron^). Consistent with previous reports (*39*), the *bgaA* deletion strain showed attenuated virulence, while mice infected with WT, Δ*bgaA*:pBgaA-T, or Δ*bgaA*:BgaA-T^ΔDegron^ succumbed to infection (Fig. 5A and fig. S12, A to D). However, the group of mice infected with the SPN strain lacking the degron sequence showed higher proportion of deaths but with delayed mortality compared to Δ*bgaA*:pBgaA-T–infected group (*P* = 0.0492, log-rank test) (Fig. 5A). Comparison of bacterial burdens in blood (Fig. 5B) and spleen (Fig. 5C) and the time course of visible disease signs in infected mice (Fig. 5D) confirmed the trend toward increased virulence in the Δ*bgaA*:pBgaA-T^ΔDegron^ strain.

Previous studies have demonstrated that SPN sepsis is established from a reservoir of bacteria in the spleen (*38*). While the first wave of invading bacteria in the circulation is rapidly cleared by host innate immune mechanisms, a proportion of SPN survive and proliferate within splenic macrophages, before reseeding into blood. We hypothesized that delayed onset of severe disease in mice infected with Δ*bgaA*:pBgaA-T^ΔDegron^ might be the result of prolonged survival of SPN within splenic macrophages, due to reduced intracellular recognition of bacteria by the host ubiquitination machinery. In support of this, we observed delayed onset of the second wave of bacteremia in mice infected with Δ*bgaA*:pBgaA-T^ΔDegron^ strain compared to Δ*bgaA*:pBgaA-T (24 hours versus 12 hours), following the early clearance phase (Fig. 5E). However, in the eclipse phase, during which bacteria are cleared from blood, splenic bacterial numbers were consistently higher in Δ*bgaA*:pBgaA-T^ΔDegron^–infected mice (Fig. 5F). These findings suggest that the phase of SPN propagation within splenic macrophages is extended in the absence of intracellular recognition of infection via the ubiquitination machinery. As a result, increased bacterial densities can accumulate in spleen (Fig. 5F), subsequently seeding into blood in higher numbers, which may account for the delayed but increased mortality of Δ*bgaA*:pBgaA-T^ΔDegron^–infected mice. Together, these data demonstrate that recognition and ubiquitination of intracellular SPN contributes to host control of pathogens during sepsis.

## DISCUSSION

Metazoans use ubiquitination as a versatile mechanism to maintain cytosolic homeostasis, preventing accumulation of damaged proteins/organelles and defending against invading pathogens (*40*, *41*). Given the variety of pathogens and diverse set of damaged proteins encountered, identification of common motifs for substrate recognition and subsequent ubiquitination represents a smart strategy for resource optimization. Proteins in eukaryotic cells that are destined for proteolysis are typically identified by a tripartite degron motif (*24*). This prompted us to explore whether similar motifs could be found on pathogen surfaces, where they might function as a proxy for substrate recognition. Here, we demonstrate that host ubiquitin ligases use similar molecular signatures to sense phylogenetically distinct pathogens. Pathogen recognition via common molecular patterns/motifs (PAMPs) is a well-characterized feature of innate immunity (*42*). Our results suggest that degron-like sequences within bacterial surface proteins act in a manner equivalent to PAMPs, to impart intracellular pathogen surveillance. This modus operando could be further exploited by the host for presentation of microbial protein antigens on major histocompatibility complex I to induce a strong CD8 response directed against intracellular pathogens. The potential importance of ubiquitin-mediated detection of pathogens to host defenses is further highlighted by the substantial residual ubiquitination detected in mutant SPN that lack both BgaA and PspA. This indicates that there are further, unidentified substrates of the ubiquitin machinery, with the resulting redundancy ensuring robust pathogen interception. Notably, some immediate questions about the degron motif and its importance to the bacteria (other than being a recognizable unit) provide an interesting evolutionary angle. Apparently, degron motif is dispensable in vivo, and if anything, mutating or deleting the motif improved intracellular persistence resulting in increased virulence. This is substantiated by our results that mutation in degron motif could be used by pathogens to escape ubiquitin-mediated recognition as seen for SPN serotype 19F. However, the conserved nature of the degron motif in BgaA across the majority of the SPN serotypes could be suggestive of a counter strategy adopted by bacteria to be less lethal to the host. This could provide an opportunity to the pathogen to increase its occupiable habitats. Concurrently, ubiquitination of the bacterial surface or secreted proteins at degron motif could modulate them functionally to dampen or rewire the host response for eventual benefit (*43*). Therefore, the presence or absence of eukaryotic-like functional domains or motifs (such as degrons) could be dependent on pathogenic niche and adapted to evade or modulate host immune responses.

A similar pathogen sensing mechanism involving guanylate-binding proteins has also been implicated in bacterial clearance; however, unlike ubiquitination, their role is reported to be restricted to Gram-negative pathogens (*44*–*47*). Ubiquitination targets the pathogen irrespective of Gram origin, for instance, RNF213 has been documented to target *Listeria* spp. irrespective of the absence of LPS (*48*). We therefore do not eliminate the possibility of its antimicrobial action against SPN too. A pathogenic surface can possibly be ubiquitinated with multiple chain types by different E3 ligases with interchain interactions (*41*). In particular, in case of Mtb, Smurf1 and Parkin have been proven to form K48 and K63 chain types that function synergistically to degrade the pathogen (*12*). Understanding the mechanistic role of K48 ubiquitination and the E3 ligases involved during infection scenario is still understudied. In case of STm, a single E3 ligase ARIH1 has been depicted to target cytosolic STm with K48 ubiquitin chains leading to their elimination from the system (*15*). On the basis of our and other’s findings, K48-Ub–labeled pathogens are lastly degraded while being associated with proteasomal machinery. However, this study shows K48-Ub decoration of specific bacterial surface proteins. Some pathogens are known to actively modify their surface proteins, protecting them from ubiquitination and subsequent killing, thus emphasizing the significance of surface localization of the ubiquitin substrate (*10*). In addition, multiple obligate intracellular pathogens have evolved strategies to de-ubiquitinate themselves, or host regulatory components, to evade ubiquitin-mediated clearance (*5*). Together, these findings underscore the importance of the ubiquitin-mediated alarm arousal as a fundamental intracellular pathogen sensing mechanism central to host defenses.

Our study further demonstrated the central role of SCF E3 ligase, particularly with FBXW7, in bacterial ubiquitination augmenting pathogen degradation. Heterozygous mutations in FBXW7, particularly R505C (a critical residue in substrate recognition pocket) variant, trigger multiple carcinomas and lymphocytic leukemia in humans (*49*, *50*). A recent cohort-based study indicated that almost 43% of the patients with chronic lymphocytic leukemia (CLL) succumb to bacterial pneumonia and sepsis, followed by fungal infections (*51*). This corroborates with our observation of the abrogated ability of host cells bearing FBXW7 mutation to sense and eliminate pathogens. Our findings therefore provide an unexpected molecular explanation of the enhanced risk of infections in patients with CLL. The link between genetic polymorphism in E3 ligase genes and susceptibility to bacterial infections is also substantiated by patients with Parkinson’s disease, who are vulnerable to typhoid fever or leprosy (*14*), suggesting noteworthy contribution of E3 ligases in host immunity against bacterial infections and maintenance of cellular steady state. In conclusion, we deciphered a universal language of sensing cytosol-dwelling pathogens that could be efficiently applied by the host to recognize microorganisms for subsequent elimination. Collectively, these findings shed light on understanding the rudimentary cellular immune processes, which could be harnessed to intensify the antibacterial immunity.

## MATERIALS AND METHODS

### Cell culture

Both the human lung alveolar carcinoma (type II pneumocyte) cell line A549 [American Type Culture Collection (ATCC) no. CCL-185)] and the cervical adenocarcinoma cell line HeLa (ATCC no. CRM-CCL-2) were cultured in Dulbecco’s modified Eagle’s medium (DMEM; HiMedia) supplemented with 10% fetal bovine serum (Gibco) at 37°C and 5% CO_2_.

### Bacterial strains and growth conditions

SPN (R6, serotype 2, gift from Tim J. Mitchell, University of Birmingham, United Kingdom) was grown in Todd-Hewitt broth supplemented with 1.5% yeast extract at 37°C in 5% CO_2_. The following antibiotics were used to grow SPN cultures when required: kanamycin (200 μg/ml), spectinomycin (100 μg/ml), and chloramphenicol (4.5 μg/ml). Nine hundred microliters of 0.4 OD_600_ (optical density at 600 nm) grown SPN culture was mixed with 600 μl of 80% sterile glycerol (32% final glycerol concentration) and stored in a −80°C deep freezer. These glycerol stocks were used as starting inoculum for all experiments.

*Escherichia coli* DH5α and STm (ATCC 14028) cultures were grown in Luria-Bertani broth (LB) at 37°C under shaking conditions (200 rpm), and when necessary, the following antibiotics were used: kanamycin (50 μg/ml), spectinomycin (100 μg/ml), chloramphenicol (20 μg/ml), and ampicillin (100 μg/ml). For all infection assays, bacteria were grown till OD_600_ ~0.4, followed by resuspension in phosphate-buffered saline (PBS) to similar density before infecting the host cells.

### Screening of putative ubiquitin target proteins

All surface proteins present in the STm and SPN proteome were first identified from published literature. Complete protein sequences of the surface proteins were obtained from UniProt, which were then used to identify the presence of degron motifs. A set of 29 putative degron motifs were curated from published literature (*24*, *52*, *53*) and the Eukaryotic Linear Motif database. The Python’s regex module was used to identify the location of all such curated motifs in the surface protein sequences. A linear search was further performed to identify the presence of a lysine residue in close proximity (8 to 14 amino acid residues) to every identified degron motif. This lysine residue is presumed to act as the attachment site for ubiquitin moiety. Last, the shortlisted protein sequences were fed into IUPred (*54*), a protein structure predicting tool, to locate the presence of disordered region in between the degron motif and proximal lysine. The resultant proteins (BgaA and PspA for SPN and RlpA for STm) were selected for evaluation as ubiquitination targets and decoding their role in pathogen clearance.

### Bacterial strain construction

Allelic exchange by homologous recombination was carried out using gene flanking regions carrying an inserted antibiotic cassette for generation of mutant strains in both SPN and STm (table S2). For SPN, 500-bp upstream and downstream regions of *bgaA*, *pspA*, and *hysA* genes were amplified from the genome with appropriate primers (table S3) and assembled in pBKS vector. Following cloning of these fragments, antibiotic resistance cassette (spectinomycin for *bgaA* as well as *pspA* and chloramphenicol for *hysA*) was inserted into this construct. The linearized recombinant plasmids were then transformed into WT SPN using competence stimulating peptide 1 (GenPro Biotech), recombinants were selected using respective antibiotic, and gene replacement was confirmed by polymerase chain reaction (PCR) and sequencing of the respective gene loci. For STm, the λ-red recombinase method was used for generation of gene deletion mutants (*55*). Briefly, sequences homologous to the ends of the *rlpA* gene were appended in the primer sequences that are used for amplification of the kanamycin resistance cassette. The cassette was then electroporated into the WT STm strain, and knockouts generated by homologous recombination were selected on the basis of kanamycin resistance. Gene deletion was confirmed using PCR and sequencing of the gene locus. Full-length *pspA*, *hysA*, and a trauncated *bgaA-T* (1 to 3168 bp) were cloned under P23 promoter in the shuttle vector pIB166 (*56*) and used for complementation. These recombinant plasmids were also used for site-directed mutagenesis to generate different variants of *bgaA-T* (*bgaA-T^K96R^*, *bgaA-T^K97R^*, *bgaA-T^K96R,K97R^*, and *bgaA-T*^Δ*Degron*^), *pspA* (*pspA^K314R^*, *pspA^K315R^*, *pspA^K314R,K315R^*, and *pspA*^Δ*Degron*^), and *hysA* (*hysA^Degron-BgaA^* and *hysA^Degron-PspA^*) using appropriate primer sets (table S3) and transformed into Δ*bgaA* and Δ*pspA* mutants. All clones were verified by DNA sequencing. Expression of different variants of BgaA, PspA, and HysA was confirmed by Western blot using appropriate antibodies.

### Antibodies and reagents

Anti-Enolase and anti-PspA serum (S. Hammerschmidt, University of Greifswald, Germany); anti-BgaA serum (S. King, The Ohio State University, USA); and antibodies specific for His_6_ (Invitrogen, MA1-21315), K48-Ub linkage (Millipore, 05-1307), K63-Ub linkage (Millipore, 14-6077-80), Ply (Santa Cruz Biotechnology, sc-80500), FBXW7 (Bethyl Laboratories, A301-721A), SKP1 (Invitrogen, MA5-15928), Cullin1 (Invitrogen, 71-8700), glyceraldehyde phosphate dehydrogenase (Millipore, MAB374), GSK3β [Cell Signaling Technology (CST), D5C5Z], phosphothreonine (CST, 9381S), IgA, Kappa from murine myeloma, clone TEPC-15 (Sigma-Aldrich, M1421), and PSMB7 (Invitrogen, PA5-111404) were procured. The following secondary antibodies were used: horseradish peroxidase (HRP)–tagged anti-rabbit (BioLegend, 406401), HRP-tagged anti-mouse (BioLegend 405306), anti-rabbit Alexa Fluor 488 (Invitrogen, A27206), anti-rabbit Alexa Fluor 555 (Invitrogen, A31572), biotin-conjugated anti-mouse immunoglobulin A (Life Technologies, M31115), anti-mouse Alexa Fluor (Invitrogen, A31570), anti-mouse Alexa Fluor 488 (Invitrogen, A21202), anti-goat Alexa Fluor 633 (Invitrogen, A21082), and FM4-64 (Thermo Fisher Scientific, T13320).

### Protein expression and purification

BgaA-T and its variants were cloned in pET28 using Xba I/Not I restriction sites. Recombinant plasmids encoding BgaA-T with N-terminal His-tag were transformed into *E. coli* BL21 (DE3) cells for protein expression. Freshly transformed colonies were grown in LB containing kanamycin (50 μg/ml) at 37°C on a shaker incubator for 12 hours. One percent of the primary culture was added to 1 liter of LB broth and incubated at 37°C on a shaker incubator till the OD_600nm_ reached between 0.6 and 0.8. Protein expression was induced by the addition of 100 μM isopropyl-β-d-thiogalactopyranoside (IPTG) and growing the culture further at 37°C for 5 to 6 hours with agitation at 150 rpm. The cells were harvested by centrifugation at 6000 rpm for 10 min at 4°C. The cell pellet was resuspended in buffer A [25 mM tris (pH 8.0) and 300 mM NaCl] and lysed by sonication. Cell debris was separated by centrifugation (14,000 rpm, 50 min, 4°C), and the supernatant was applied onto a Ni-NTA column, equilibrated with buffer A. The column was washed with 10 column volumes of buffer A, and the His-tagged proteins were eluted with imidazole (250 mM) in buffer A. All the variants of BgaA-T were expressed and purified using the same procedure. FBXW7 was subcloned in pGEX-4 T-1 vector having an N-terminal glutathione *S*-transferase (GST) tag from pCMV6-Entry-FBXW7 vector using Eco RI restriction enzyme. GST-tagged FBXW7 was expressed in *E. coli* BL21 (DE3) cells following induction of protein expression with 0.1 mM IPTG and growth at 30°C for 4 hours. GST-FBXW7 from *E. coli* crude extract was purified using Glutathione-Sepharose (GE HealthCare) column chromatography. Fractions containing purified proteins were pooled and concentrated up to 0.5 mg/ml using a 10-kDa molecular weight cutoff filter (Amicon) by centrifugation at 4700 rpm at 4°C. Purity of the proteins was checked on SDS–polyacrylamide gel electrophoresis (SDS-PAGE) followed by staining with Coomassie blue.

### Host cell transfections

BgaA-T was cloned in doxycline-inducible vector pAK_Tol2_TRE_Blast (Addgene no. 130261) using in-fusion cloning kit (Takara). A positive clone was confirmed by Sanger sequencing. FBXW7^R505C^ mutation was carried out by site-directed mutagenesis using pMRX-GFP-FBXW7 as template and confirmed by Sanger sequencing. All transfections were performed using Lipofectamine 3000 reagent (Thermo Fisher Scientific), and selections were done in the presence of blasticidin hydrochloride (2 μg/ml; HiMedia).

### siRNA-directed gene knockdown

For RNA interference–mediated knockdown of specific genes, the following siRNAs were used: siFBW7 (Dharmacon ON-TARGET SMARTpool L-004246-00-0005), siCullin1 (Qiagen, 1027423), siSKP1 (Qiagen, SI00301819), and siGSK3β (Dharmacon ON-TARGET SMARTpool L-003010-00-0005). Briefly, A549 cells grown in a 24-well plate were transiently transfected with gene-specific siRNAs or scramble (siControl) (150 pmol) using Lipofectamine 3000 as per the manufacturer’s instructions. Thirty-six hours after transfection, cells were processed for Western blotting and immunofluorescence or penicillin-gentamycin protection assays.

### Structure prediction and modeling

All structures were predicted by AlphaFold (Protein Homology/analogY Recognition Engine V 2.0) (*57*) and visualized using PyMOL (The PyMOL Molecular Graphics System, version 2.0 Schrödinger, LLC). Structures were color coded in PyMol based on IUPred (*54*) scores ranging from ordered (blue) to disordered (red) through white.

### Western blotting

SPN cultures grown to 0.4 OD_600nm_ were lysed by sonication, and crude extracts were collected following centrifugation (15,000 rpm, 30 min, 4°C). For A549s, monolayers were washed several times with PBS and lysed in ice-cold radioimmunoprecipitation assay (RIPA) buffer [50 mM tris-Cl (pH 7.89), 150 mM NaCl, 1% Triton X-100, 0.5% sodium deoyxycholate, and 1% SDS] containing protease inhibitor cocktail (Promega), sodium fluoride (10 mM), and EDTA (5 mM). The cell suspension was briefly sonicated and centrifuged to collect cell lysates. Proteins present in bacterial or A549 cell lysates (10 or 20 μg) were separated on 12% SDS-PAGE gels and transferred to an activated polyvinylidene difluoride membrane. Following blocking in 5% skimmed milk, the membranes were probed with appropriate primary and HRP-tagged secondary antibodies. The blots were lastly developed using an enhanced chemiluminescence substrate (Bio-Rad).

### Penicillin-gentamicin protection assay

SPN strains grown until OD_600nm_ 0.4 in Todd-Hewitt broth supplemented with 0.2% yeast extract (THY) were pelleted, resuspended in PBS (pH 7.4), and diluted in assay medium for infection of A549 monolayers with multiplicity of infection (MOI) of 10. Following 1 hour of infection, the monolayers were washed with DMEM and incubated with assay medium containing penicillin (10 μg/ml) and gentamicin (400 μg/ml) for 2 hours to kill extracellular SPN. Cells were then lysed with 0.025% Triton X-100, and the lysate was plated on Brain Heart Infusion agar plates to enumerate viable SPN. Percentage invasion was calculated as [colony-forming units (CFU) in the lysate/CFU used for infection] × 100. To assess the intracellular survival, at 9 hours after infection (from the beginning of penicillin-gentamycin treatment), cell lysates were prepared as mentioned above, and spread plated and surviving bacteria were enumerated. Survival efficiency (%) was represented as fold change in percent survival relative to control at indicated time point (normalized to 0 hours).

### Immunofluorescence

For immunofluorescence assay, A549 or HeLa cells were grown on glass coverslips and infected with SPN or STm strains at MOI ~25 for 1 hour followed by antibiotic treatment for 2 hours. At desired time points after infection (9 hours for SPN infection in A549s and 3 hours for infection with STm in HeLa), cells were washed with DMEM and fixed with ice-chilled methanol at −20°C for 10 min. Further, the coverslips were blocked with 3% bovine serum albumin (BSA) in PBS for 2 hours at room temperature (RT). Cells were then treated with an appropriate primary antibody in 1% BSA in PBS overnight at 4°C, washed with PBS, and incubated with suitable secondary antibody in 1% BSA in PBS for 1 hour at RT. Last, coverslips were washed with PBS and mounted on glass slides along with VECTASHIELD with or without 4′,6-diamidino-2-phenylindole (Vector Laboratories) for visualization using a laser scanning confocal microscope (LSM 780, Carl Zeiss) under 40× or 63× oil objectives. The images were acquired after optical sectioning and then processed using ZEN lite software (version 5.0). Superresolution microscopy was performed similarly using Elyra 7 (Carl Zeiss) in SIM mode. For colocalization analysis, bacteria were scored by visual counting of *n* > 100 bacteria per replicate.

### Transmission electron microscopy

Following infection with SPN and STm, cells were washed with 0.1 M sodium cacodylate buffer, fixed with 2.5% glutaraldehyde (Sigma-Aldrich) in 0.1 M sodium cacodylate buffer (Sigma-Aldrich) for 3 hours at 4°C. After fixation, cells were collected through a cell scraper and pelleted at 2000 rpm for 10 min. Cells were then washed with 0.1 M sodium cacodylate buffer and postfixed with 1% osmium tetroxide in 0.1 M sodium cacodylate buffer for 20 min at 4°C. Following subsequent washes with cacodylate buffer and distilled water, cells were then dehydrated in increasing concentrations of ethanol (50, 70, 95, and 100%) and propylene oxide for 30 min and lastly embedded in epoxy resin (Electron Microscopy Sciences, 14300) by polymerization at 75°C for 45 min. Subsequently, the capsules were transferred to a 95°C oven for 45 min. Ultrathin sections (70 nm) were cut using a diamond knife on a Leica EM UC7 Ultramicrotome and stained with 2% uranyl acetate and 1% lead citrate and viewed using a transmission electron microscope (Talos L120 C, Thermo Fisher Scientific) at 120 KV.

### Ex vivo ubiquitination

A549-expressing BgaA-T and its variants were treated with MG132 (5 μM), and protein expressions were induced with doxycline (100 μg/ml) for 24 hours. Cells were then lysed in RIPA buffer, and samples were electrophoresed by SDS-PAGE (8%). Confirmation of protein expression and ubiquitination of BgaA-T and its variants were proved by Western blot following probing with anti-BgaA and anti–K48-Ub antibodies, respectively.

### In vitro ubiquitination

For in vitro ubiquitination assays, recombinant proteins were purified as described earlier (*58*). The SCF^FBW7^ E3 ligase complex was incubated with recombinant 0.1 mM E1 (UBE1; Boston Biochem), 0.25 mM E2 (cdc34; Boston Biochem), and ubiquitin (2.5 μg/ml; Boston Biochem) in the presence of purified BgaA-T and its variants. Ubiquitylation reactions were performed in assay buffer [50 mM tris (pH 8), 5 mM MgCl_2_, 5 mM adenosine triphosphate (ATP), 1 mM β-mercaptoethanol, and 0.1% Tween 20] for 2 hours at 25°C. The reactions were stopped with 5× Laemmli buffer, resolved on SDS-PAGE gels, and analyzed by immunoblotting using an anti-BgaA antibody.

### In vitro kinase assay

To perform in vitro kinase assay, GSK3β kinase enzyme system (Promega) was used as per the manufacturer’s protocol with following modifications. Briefly, 3 μg of recombinant BgaA-T was added to 0.5 μg of active GSK3β with 400 μM ATP in reaction buffer composed of 40 mM tris, (pH 7.5), 20 mM MgCl_2_, BSA (0.1 mg/ml), and 50 μM dithiothreitol. The reaction was incubated for 2 hours at 30°C and stopped by adding 1× Laemmli buffer. The samples were then boiled and electrophoresed for Western blotting as mentioned previously. Phosphorylated BgaA-T was detected with anti-phosphothreonine antibody.

### In vivo model

All animal experiments were performed at the University of Liverpool in strict accordance with U.K. Home Office guidelines, under project license PP2072053, following approval from local animal welfare and ethics committees. For infection studies, 7- to 8-week female CD1 mice were purchased from Charles River Laboratories, United Kingdom and allowed to acclimatize for 7 days before use. Briefly, mice were placed into a restraint tube, and *S. pneumoniae* was administered by intravenous injection into the tail vein (1 × 10^6^ CFU in 100 μl of PBS). Mice were periodically scored for clinical signs of disease and culled when they showed signs of advanced pneumococcal disease or else at predetermined times after infection. Severity endpoints were defined as one or more of the following: substantially elevated or reduced respiratory rate, substantial reduction in natural behavior or moderate reduction in provoked behavior, loss of >20% starting body weight, and pronounced nasal or ocular discharge. Blood samples were obtained by cardiac puncture under terminal anesthesia, and spleens were excised postmortem for bacterial enumeration. Tissue samples were processed with a hand-held tissue homogenizer, and homogenates were serially diluted in PBS before spotting onto blood agar plates. Plates were incubated overnight at 37°C, 5% CO_2_, and bacterial colony numbers were assessed the following day.

### Statistical analysis

GraphPad Prism version 5 was used for statistical analysis. Statistical tests undertaken for individual experiments are mentioned in the respective figure legends. *P* < 0.05 was considered to be statistically significant. Data were tested for normality and to define the variance of each group tested. All multiparameter analyses included corrections for multiple comparisons, and data are presented as means ± SD unless otherwise stated.

## Notes

View/request a protocol for this paper from *Bio-protocol*.
