# Supplementary material for: An innate pathogen sensing strategy involving ubiquitination of bacterial surface proteins
Source: Sci Adv. 2023 Mar 22;9(12):eade1851. doi: 10.1126/sciadv.ade1851 (PMC10032600; doi:10.1126/sciadv.ade1851)
Supplement: Supplementary file 1 — Figs. S1 to S12 Tables S1 to S3 [file sciadv.ade1851_sm.pdf]

Science Advances
AAAS

Supplementary Materials for

An innate pathogen sensing strategy involving ubiquitination of bacterial surface proteins

Shruti Apte *et al.*

Corresponding author: Anirban Banerjee, abanerjee@iitb.ac.in

Sci. Adv. **9**, eade1851 (2023)
DOI: 10.1126/sciadv.ade1851

This PDF file includes:

Figs. S1 to S12
Tables S1 to S3

Fig.S1: Cytosolic SPN and STm are predominantly K48-Ub positive.

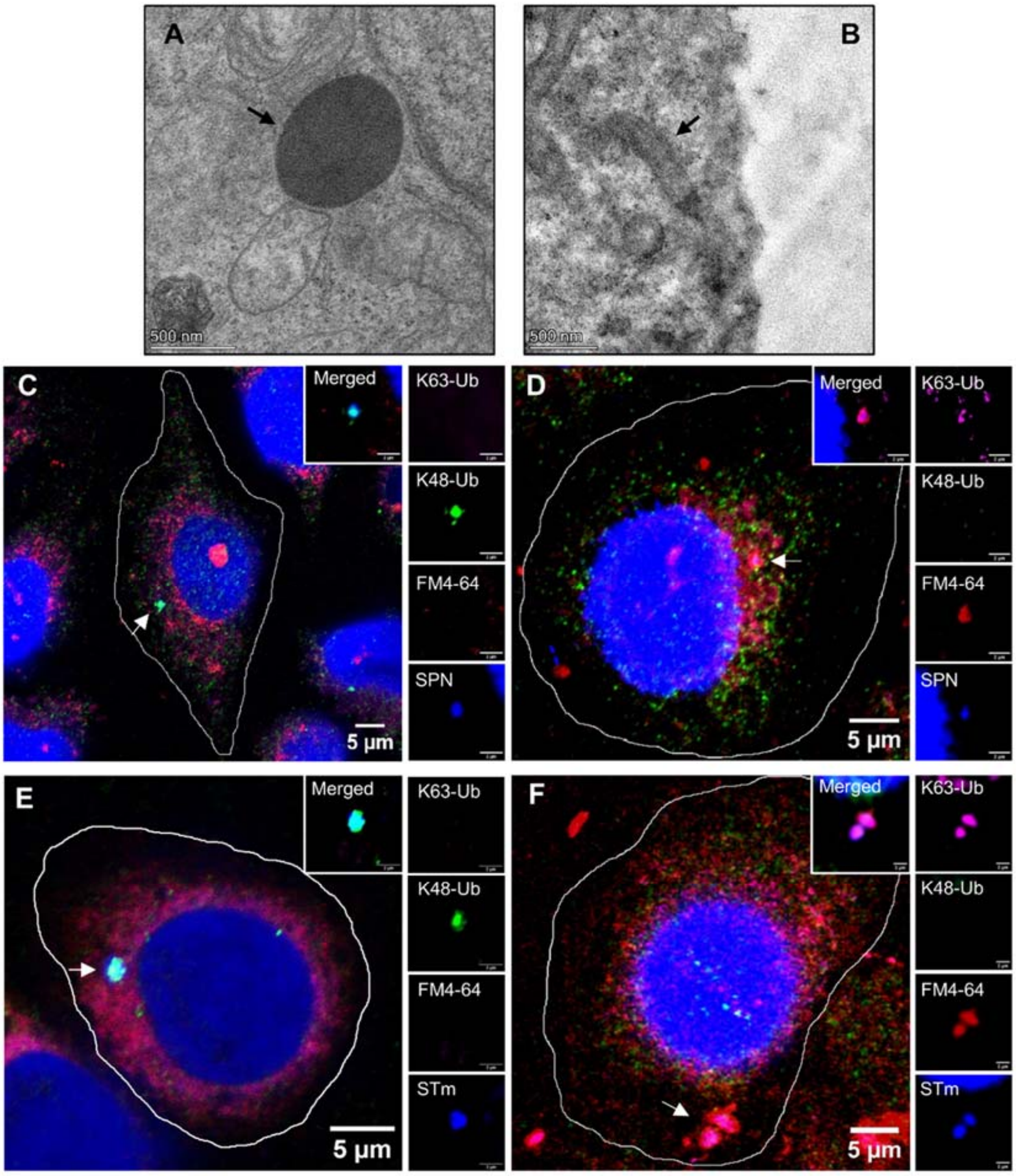

A-B. Transmission electron micrograph depicting cytosolic presence of WT SPN (**A**) and STm (**B**) in A549 and HeLa cells, respectively. Scale bar, 0.5 µm. **C-F.** Representative immunofluorescence images showing association of SPN or STm with membrane marker, FM4-64 and K48-Ub and K63-Ub chains. SPN^{+}-FM4-64^{-}-K48-Ub^{+}-K63-Ub^{-} **(C)**, SPN^{+}-FM4-64^{+}-K48-Ub^{-}-K63-Ub^{+} **(D)**, STm^{+}-FM4-64^{-}-K48-Ub^{+}-K63-Ub^{-} **(E)**, STm^{+}-FM4-64^{+}-K48-Ub^{-}-K63-Ub^{+} **(F).** Scale bar, 5 µm.

Fig.S2: Predicted structures of ubiquitination targets.

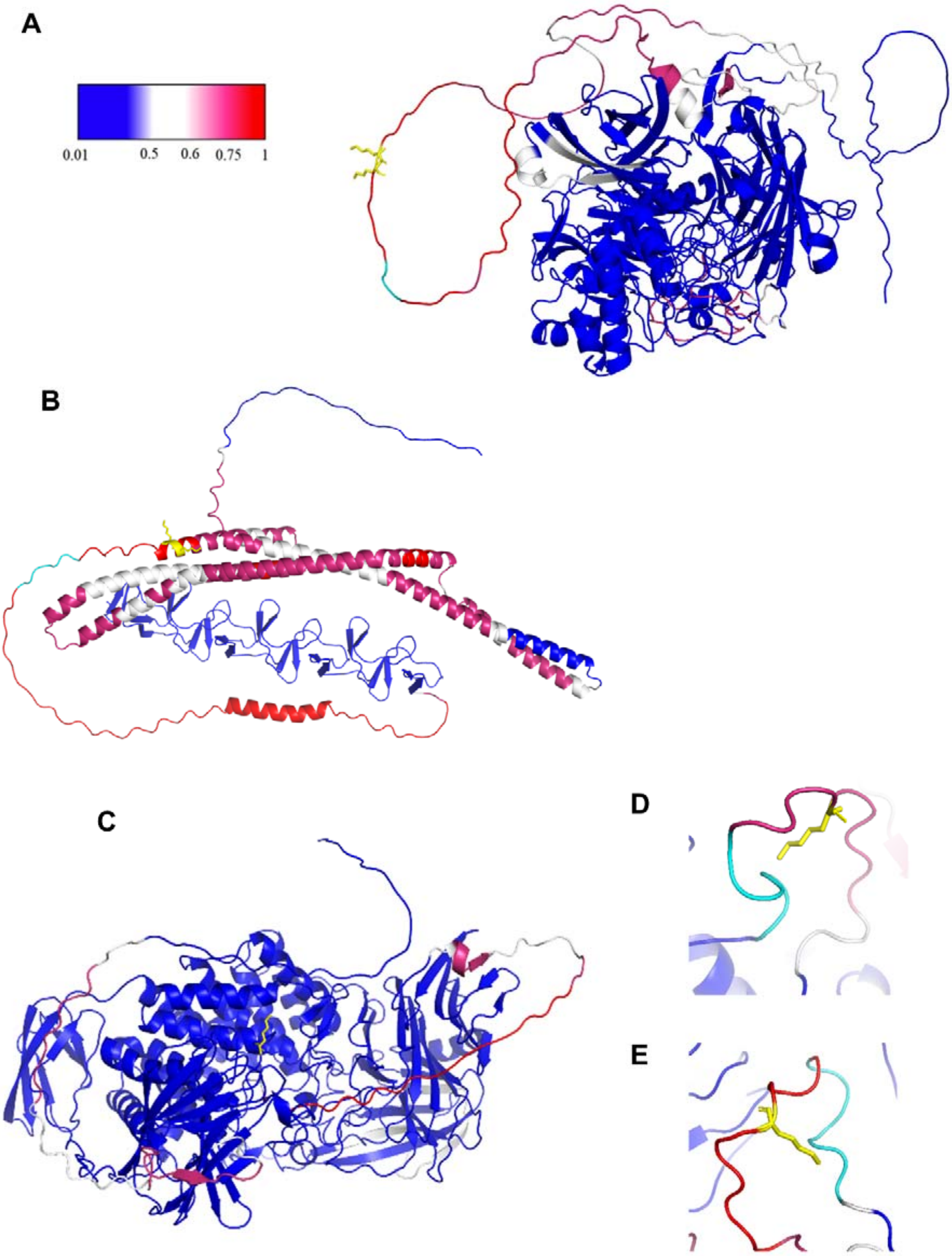

A-C. Structures of BgaA (**A**), PspA (**B**) and HysA (**C**) as predicted by Alpha-fold and visualized using PyMOL. Shown also are zoomed in areas of the protein containing the degron sequence and the lysine residue tagged for ubiquitin attachment. **D-E.** Zoomed in version of predicted HysA structures following incorporation of the degron sequence of BgaA (**D**) or PspA (**E**) into HysA. Color coding is based on scores predicted by IUPred, ranging from ordered (blue) to disordered (red) through white where cyan depicts a degron region while lysine residue is shown in yellow ball and stick conformation. The scheme with respective IUPred scores and its color coding is shown.

Fig.S3: Degron deletion from BgaA does not alter the growth and adherence of SPN as well as the structure of the protein.

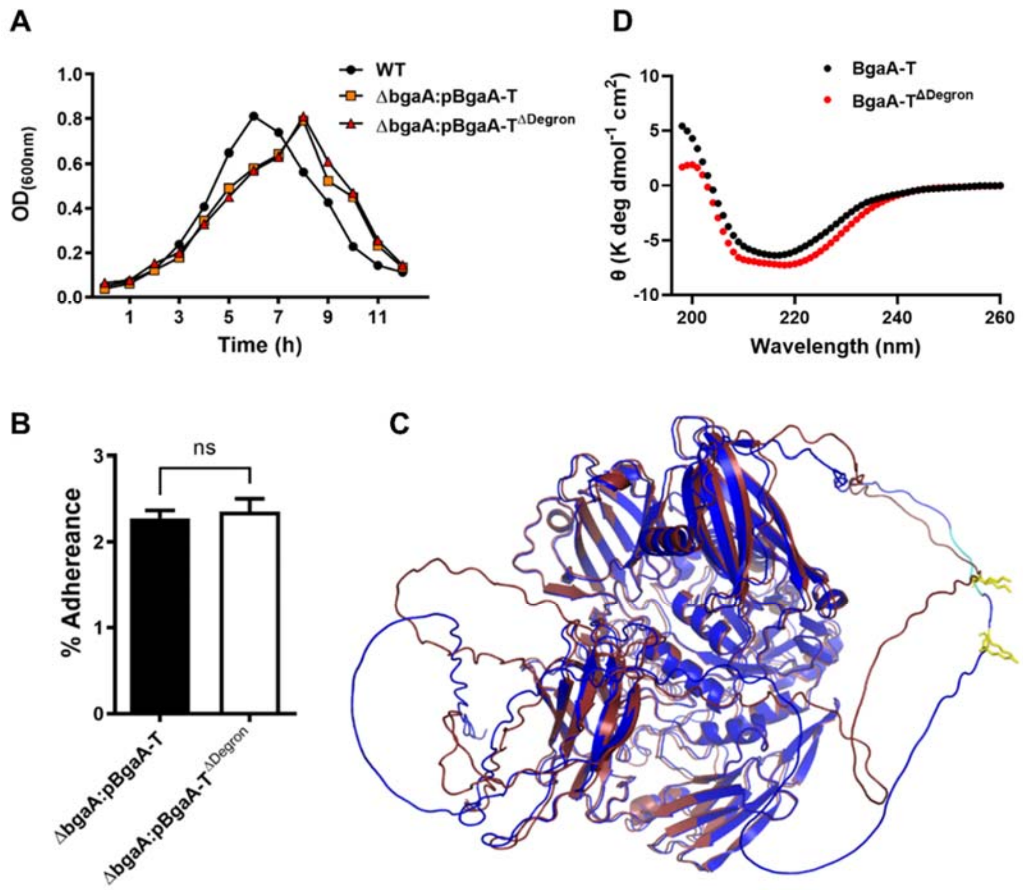

A. Growth profile of WT, Δ*bgaA*:pBgaA-T and Δ*bgaA*:pBgaA-T$^{\Delta Degron}$. **B.** Percent adherence of Δ*bgaA*:pBgaA-T and Δ*bgaA*:pBgaA-T$^{\Delta Degron}$ strain to A549s. **C.** Superimposed structures of BgaA-T and BgaA-T$^{\Delta Degron}$ as predicted by Alpha-fold and visualized using PyMOL. BgaA-T is color coded in blue with cyan depicting a degron motif while BgaA-T$^{\Delta Degron}$ is shown in red. In both cases, the lysine residues are marked in yellow in ball and stick configuration. **D.** CD spectroscopic analysis showing similar secondary structures of BgaA-T and BgaA-T$^{\Delta Degron}$. Statistical significance was assessed by two-tailed unpaired student's t-test. ns = non-significant. Data are mean ± SD of 3 independent biological replicates.

Fig.S4: PspA is also a target for ubiquitination.

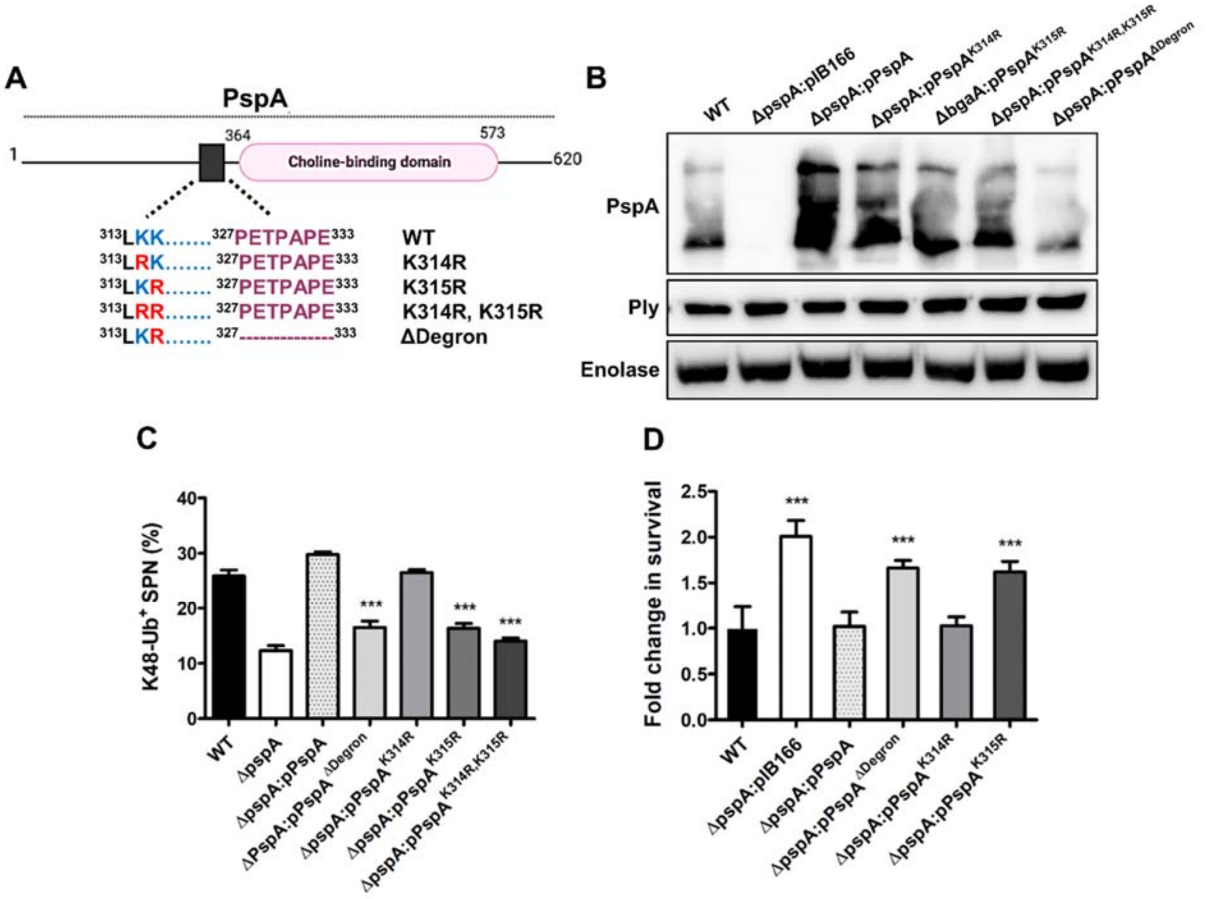

A. Schematic diagram of PspA depicting presence of degron and proximal lysine residues. Also shown are different mutant PspA variants used in the study. **B**. Immunoblot showing expression of PspA variants in Δ*pspA* strains. All strains expressed similar levels of pneumolysin (Ply) ensuring equal damage to endosome membrane. Enolase was used as a loading control. **C**. Percentage of decoration of different SPN strains with K48-Ub at 9 h post infection in A549 cells. n>100 bacteria/coverslip. **D**. Fold change in intracellular survival of different SPN strains carrying mutations in the putative lysine residues or deletion of degron sequence, in A549s normalized to WT SPN at 9 h post infection. Statistical significance was assessed by one-way ANOVA followed by Dunnett's test (C, D). ***P<0.005. Data are mean ± SD of 3 independent biological replicates.

Fig.S5: Mutation in degron motif of BgaA dampens its recognition by host ubiquitination machinery.

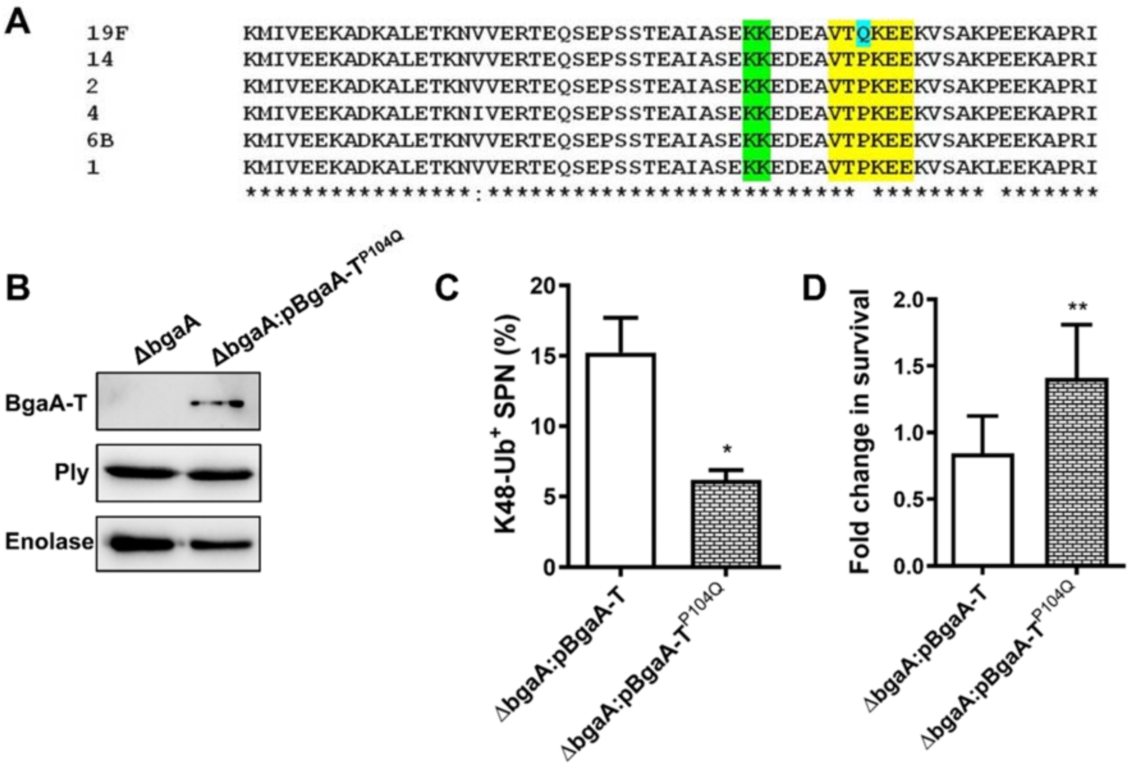

A. Sequence alignment of tripartite degron region in BgaA of different pneumococcal serotypes. Accession numbers of BgaA in different SPN serotypes are as follows: ACF55744 (19F); ACB89855 (14); AAK99369 (2); AAK74795 (4); ADM90775 (6B); ACO20620 (1). Sequences were retrieved from KEGG genomic database and aligned in Clustal Omega. Mutation at 104th position in serotype 19F (P104Q) is highlighted in cyan. Degron sequence is highlighted in yellow and upstream lysine residues are marked in green. **B.** Immunoblot demonstrating expression of BgaA-T in Δ*bgaA* and Δ*bgaA*:pBgaA-T^{P104Q}. Ply expression ensures vacuolar damage while enolase acts as a loading control. **C, D.** Impaired recognition of Δ*bgaA*:pBgaA-T^{P104Q} strain by K48 ubiquitination machinery **(C)**, resulting in improved intracellular survival advantage **(D)** compared to Δ*bgaA*:pBgaA-T strain. Statistical significance was assessed by two-tailed unpaired student's t-test. *P < 0.05, **P < 0.01. Data are mean ± SD of 3 independent biological replicates.

Fig.S6: Association of SPN with ubiquitination machinery.

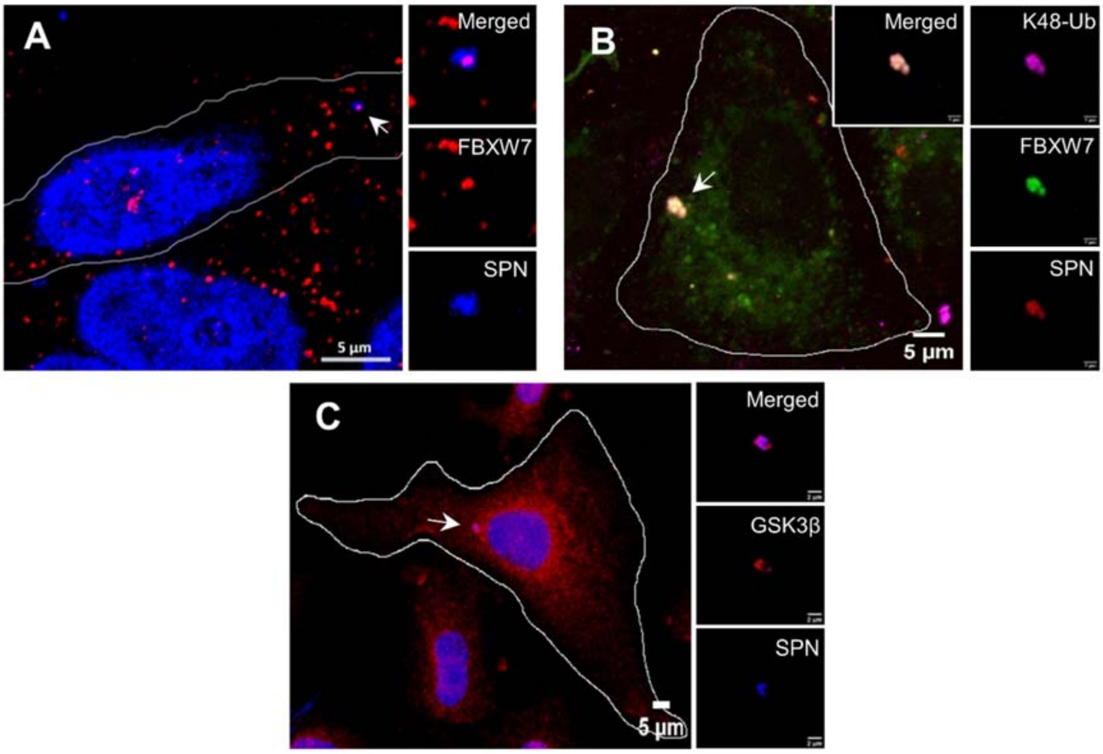

A-C. Representative confocal micrographs of FBXW7 positive SPN **(A)**, FBXW7 and K48-Ub positive SPN **(B)** and SPN associated with GSK3β **(C)**. Scale bar, 5 µm (**A** and **C**) and 10 µm (**B**).

Fig.S7: siRNA mediated gene knock-down and effect of FBXW7 mutation.

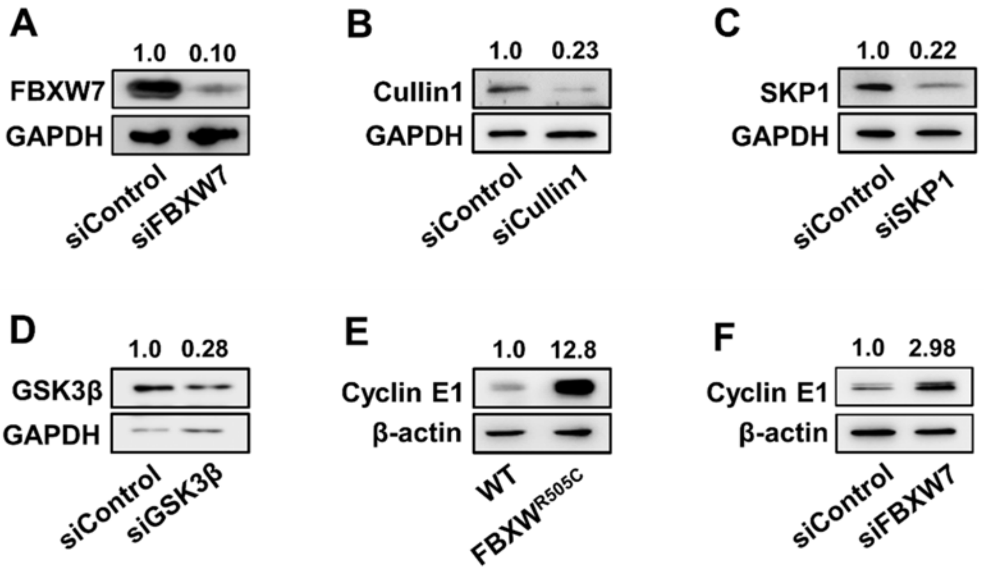

A-D. Immunoblot demonstrating knock-down in expression of FBXW7 (**A**), Cullin1 (**B**), SKP1 (**C**), the key components of SCF^{FBW7} E3-ligase, as well as GSK3β (**D**) in A549 cultures following transfection with siFBXW7, siCullin1, siSKP1 and siGSK3β, respectively. GAPDH served as loading control. Fold change in expression of individual targets relative to siControl (post normalization with GAPDH) is mentioned above the blot. Immunoblot showing accumulation of Cyclin E1 in $FBXW7^{R505C}$ cells **(E)** and siFBXW7 treated cells **(F)** compared to WT cells and siControl treated cells, respectively. β-actin served as loading control. Fold change in accumulation of Cyclin E1 in **(E)** and **(F)** (post normalization with β-actin) are mentioned above the blot.

Fig.S8: K48-ubiquitination and survival ability of mutant SPN strains following knock-down of FBXW7 and GSK3β.

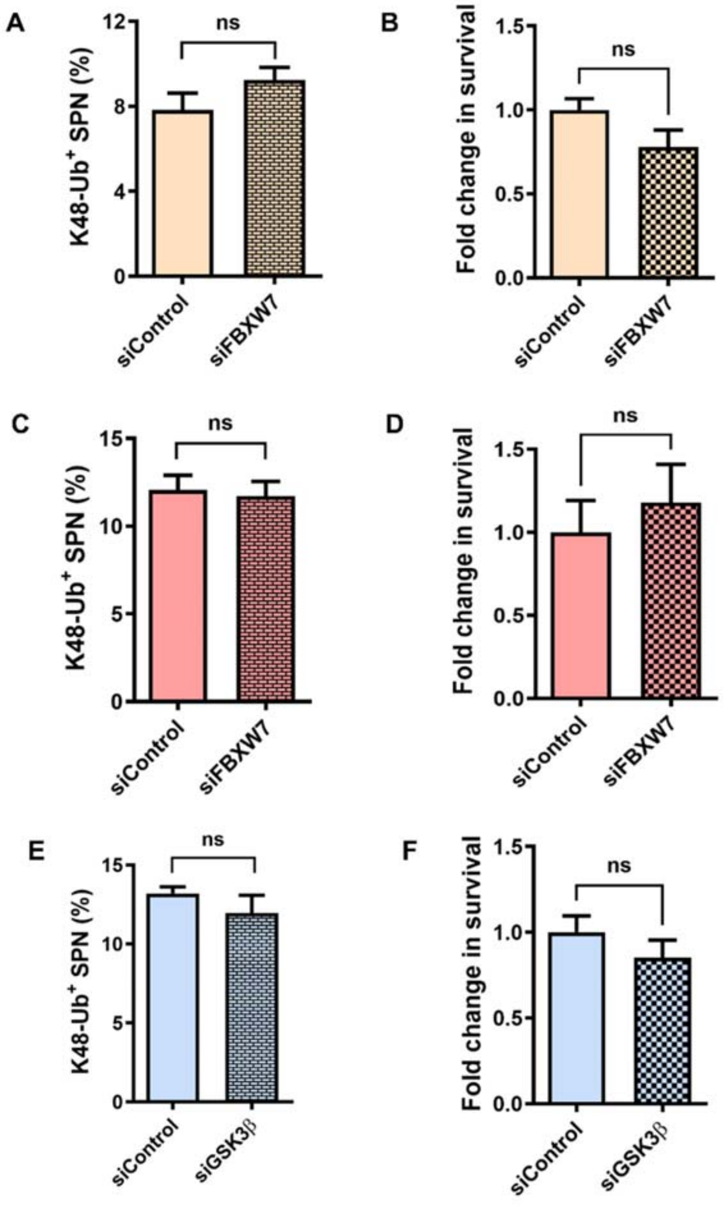

A, C. Percent association of Δ*pspA*Δ*bgaA* (**A**), Δ*bgaA*:pBgaA-$T^{\Delta Degron}$ (**C**) with K48-Ub in siFBXW7 treated cells as compared to siControl. **B, D.** Survival efficiency of Δ*pspA*Δ*bgaA* (**B**), Δ*bgaA*:pBgaA-$T^{\Delta Degron}$ (**D**) upon FBXW7 silencing. **E.** Percent association of Δ*bgaA*:pBgaA-T^{T103A} with K48-Ub following siGSK3β treatment. **F.** Survival efficiency of Δ*bgaA*:pBgaA-$T^{\Delta T103A}$ in siGSK3β treated cells as compared to siControl. Statistical significance was assessed by two-tailed unpaired student's t-test. ns = non-significant. Data are mean ± SD of 3 independent biological replicates.

Fig.S9: Purified BgaA-T variants.

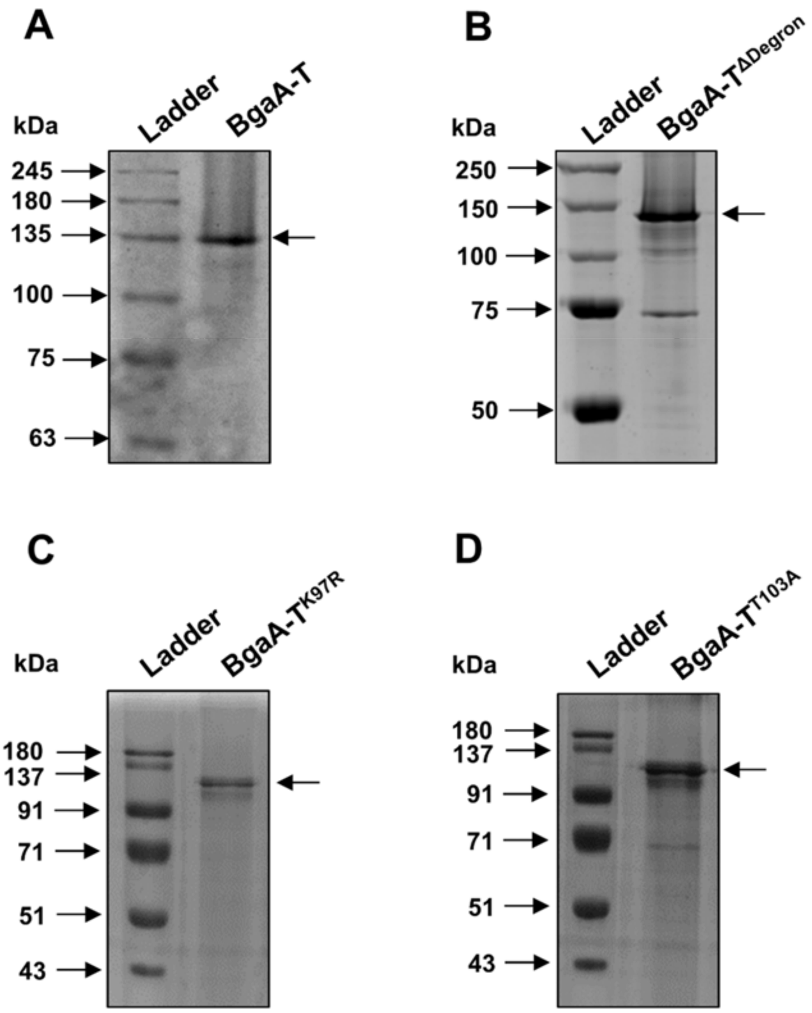

A-D. SDS-PAGE analysis of BgaA-T variants, BgaA-T (**A**), BgaA-T$^{\Delta Degron}$ (**B**), BgaA-T^{K97R} (**C**) and BgaA-T^{T103A} (**D**), expressed as His-tagged protein and purified via Ni-NTA column chromatography for use in *in vitro* ubiquitination and *in vitro* kinase reactions. Arrows by the side of gels depicts purified protein bands.

Fig.S10: PspA is phosphorylated for subsequent ubiquitination.

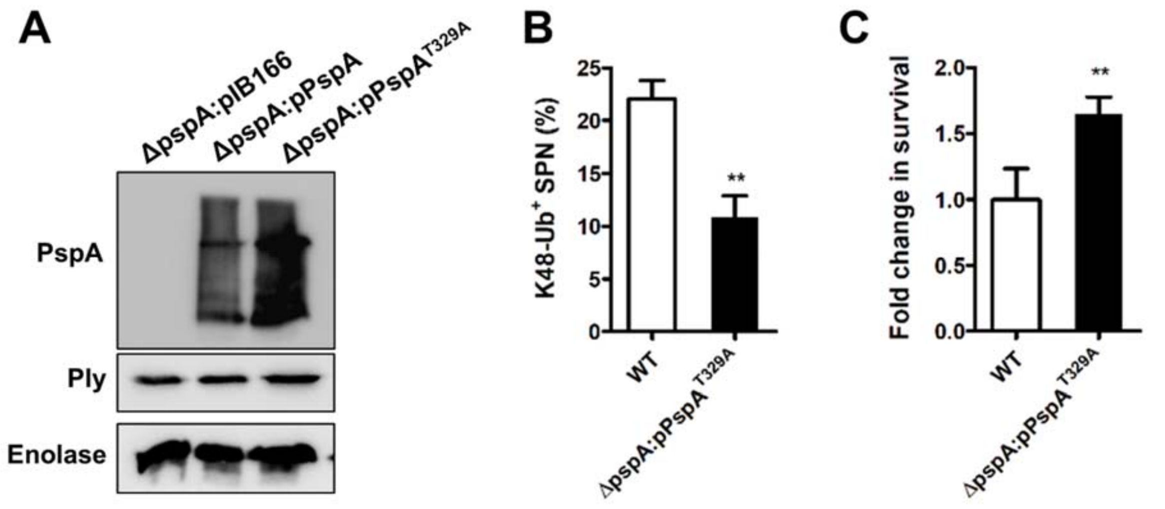

A. Immunoblot analysis of PspA complemented phosphodegron mutant variants. **B**. Percentage of K48-Ub association with different SPN strains harboring PspA variants in A549 cells at 9 h post infection. n>100 bacteria/coverslip. **C.** Fold change in intracellular persistence ability of phosphorylable and non-phosphorylable PspA variant harboring SPN strains in A549s normalized to WT SPN at 9 h post infection. Statistical significance was assessed by two-tailed unpaired student's t-test. **P < 0.01. Data are mean ± SD of 3 independent biological replicates.

Fig.S11: K48-ubiquitination directs pathogens towards proteasomal machinery.

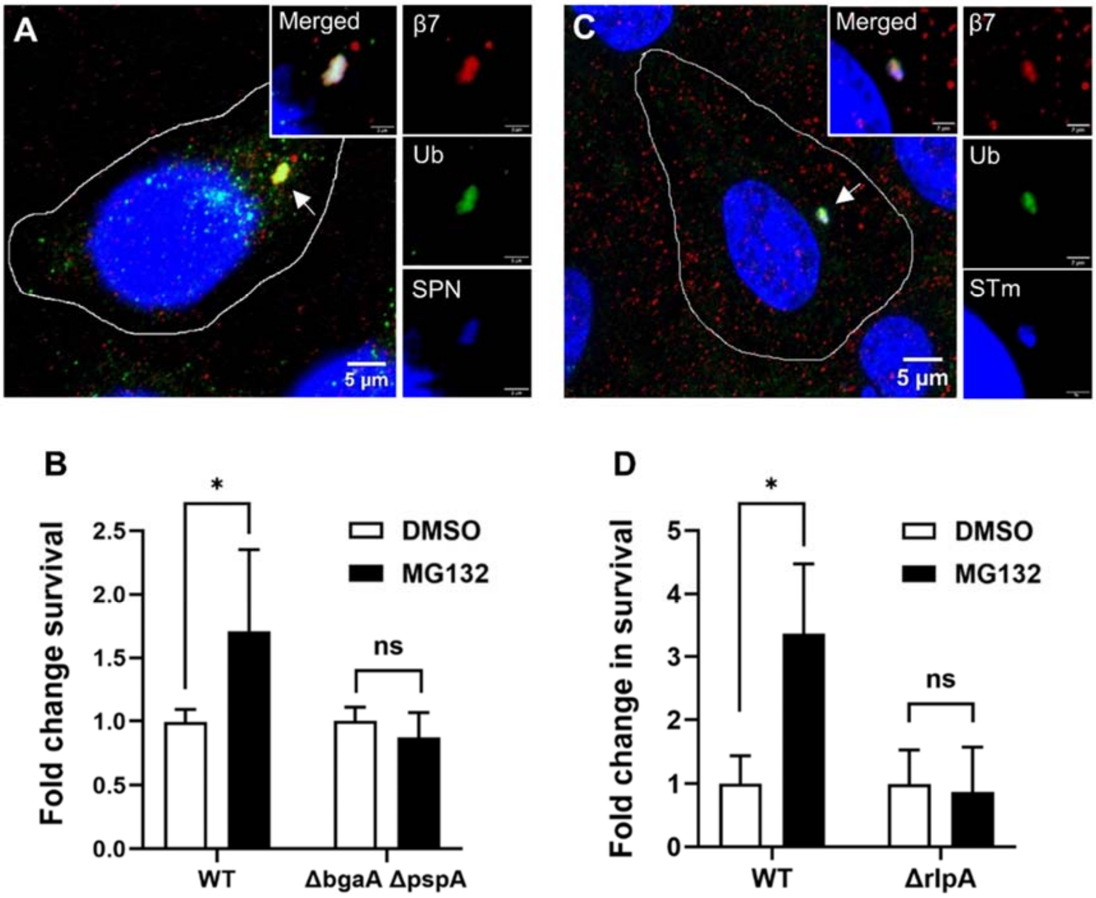

A, C. Representative confocal micrograph demonstrating association of proteasomal subunit, β7 with K48 ubiquitinated SPN **(A)** and STm **(C)**. **B**. Fold change in survival efficiency of WT and Δ*bgaA*Δ*pspA* in MG132 treated A549s as compared to non-treated cells at 9 h post infection. **D.** Fold change in intracellular persistence ability of STm and Δ*rlpA* upon MG132 treatment to HeLa cells. Statistical significance was assessed by two-tailed unpaired student's t-test. *P < 0.05; ns = non-significant. Data are mean ± SD of 3 independent biological replicates.

Fig.S12: Contribution of BgaA to SPN virulence.

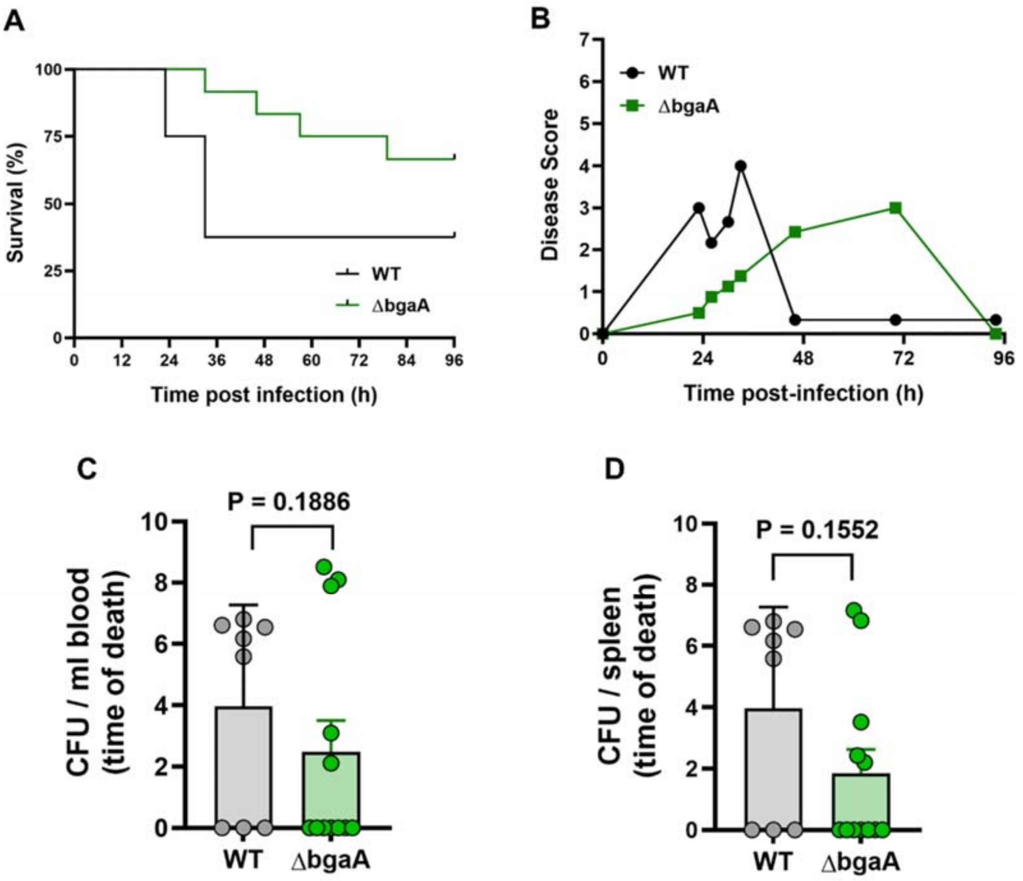

Mice were infected intravenously with 1 x 10^6 colony forming units (cfu) of wild type D39 or *bgaA* deletion strains in 50 µl PBS. **A.** Survival following intravenous infection. N=8 for wild type and N=12 for Δ*bgaA*. Kruskal-Wallis comparison of survival curves identified a significant difference between groups (p=0.0492). **B.** Physical signs of disease in infected mice. Scoring system takes into account physical appearance, weight, breathing rate and natural and provoked behaviour**. C-D.** Spleen (**C**) and blood (**D**) cfu at time of death following infection with WT and Δ*bgaA* mutant. Statistical analysis was done using unpaired student's t-test with Welch's correction and P-values obtained are mentioned above the graph.

Table S1. List of putative bacterial substrates for K48 ubiquitination.

**Uniprot ID**	**Gene name**	**Primary and secondary degron**
Streptococcus pneumoniae		
Q59947	*iga1*	^{367}**K**EEVSREI***VSTST***^{379}
Q8CZ52	*spr0440*	^{115}**K**ASEV***VAETPSAE***^{127}
Q8DRL6	*strH*	^{1141}**K**GDESG***LAPTTEVKPRLDI***^{1159}
Q8DRA6	*pulA*	^{96}**K**PATE***PTTST***^{105}
Q8CYK3	*spr1345*	^{136}**K**PTPE***PGTPKTE***^{147}
Q8DQP4	*bgaA*	^{96}**KK**EDEA***VTPKEE***^{107}
Q8CYI8	*spr1403*	^{894}**K**GAQGQAGRDG***VTPTVT***^{910}
Q8DN05	*pspC*	^{263}KRGVPGELATPD***KKEND***^{279}
Q8DRI0	*pspA*	^{314}**KK**AVNEPE**K**PAPA***PETPAPE***^{333}
***Salmonella enterica* serovar Typhimurium**		
Q8ZLK3	*damX*	^{254}**K**QAVIEPKKPQTT***AKTTT***^{271}
		^{282}**K**RTEPAA***PAATPKAT***^{296}
Q8ZR01	*rlpA*	^{229}**K**SDDTTGA***PVSSS***^{241}
Q56026	*sipD*	^{54}**K**IHQAQQT***LQSTPPIS***^{69}
Q7CPS1	*ygiB*	^{181}**K**TAMAPKP***ATTTT***^{193}<br>^{205}**K**QSTMQRSA***AGTST***^{218}
Listeria monocytogenes		
A0A3Q0NAQ4	LMON_0157	^{493}**K**F**K**ISTEALNVTVT***KENT***^{510}
A0A3Q0NHX2	Peptidoglycan hydrolase	^{289}**K**LNDLISRYNLTQYD***SGKTT***^{308}<br>^{361}**K**SDFIYPGQ**K**LKVS***AGSTT***^{379}<br>^{436}**K**SDFIYPGQ**K**LKVS***AGSTS***^{454}<br>^{510}**K**SDFIYPGQ**K**LKVS***AGSTT***^{528}
A0A3Q0NC23	Peptidoglycan bound protein	^{595}**K**PIDGFQ**K**EKPHV***VKENA***^{612}<br>^{1526}**K**VTKTT***PKDTPKDT***^{1539}
A0A3Q0NE36	Internalin-like protein	^{507}KETPI***PDPTPTPT***PD***PTPTPDPS***^{529}
A0A3Q0NBR5	Internalin-like protein	^{553}**K**AD**K**TSLT***TKENK***^{565}
A0A3Q0NEI3	Peptidoglycan bound protein	^{363}**K**PIINPVKPVN***PATST***^{378}
A0A3Q0NB40	Serine protease, *degP/htrA*	^{19}**K**REVEDTLHTPESAQP***VQETPIVE***G***VTPEGE***^{49}
Shigella flexneri		
Q54151	*pic*	^{733}**K**NDGTGNYVIP***EEGTSVPD***^{751}

Degron motif is depicted in bold and italicized font and the proximal lysine residue tipped for ubiquitination is marked in bold font.

Table S2. List of bacterial strains.

STRAIN	SOURCE
Streptococcus pneumoniae R6 (Serotype 2)	Prof. TJ Mitchell, Univ. of Birmingham, UK
SPN Δ*bgaA*	This study
Δ*bgaA*:pIB166	This study
Δ*bgaA*:pBgaA-T	This study
Δ*bgaA*:pBgaA-T^{K96R}	This study
Δ*bgaA*:pBgaA-T^{K97R}	This study
Δ*bgaA*:pBgaA-$T^{K96R,K97R}$	This study
Δ*bgaA*:pBgaA-$T^{\Delta Degron}$	This study
Δ*bgaA*:pBgaA-T^{T103A}	This study
Δ*bgaA*:pBgaA-T^{P104Q}	This study
Δ*bgaA*:pHysA	This study
Δ*bgaA*:$pHysA^{Degron\text{-}BgaA}$	This study
SPN Δ*pspA*	This study
Δ*pspA*:pIB166	This study
Δ*pspA*:pPspA	This study
Δ*pspA*:$pPspA^{K314R}$	This study
Δ*pspA*:$pPspA^{K315R}$	This study
Δ*pspA*:$pPspA^{K314R,K315R}$	This study
Δ*pspA*:$pPspA^{\Delta Degron}$	This study
Δ*pspA*:$pPspA^{T329A}$	This study
Δ*pspA*:$pHysA^{Degron\text{-}PspA}$	This study
Salmonella Typhimurium (STm) ATCC 14028	ATCC
STmΔ*rlpA*	This study

Table S3. List of primers.

NAME	TYPE	PRIMER SEQUENCE
BgaA-Upstream	F	5′-TCATATTCTAGAGGTGTAGGTGCCTTCCCAGA-3′
	R	5′-TAAAGTGGATCCAAAACCCTCCTTATATTATATTTAGTG-3′
BgaA-Downstream	F	5′-ACGGTGGGATCCAAATTTTGATACCTTCTTTATCATT-3′
	R	5′-TAAAGTCTCGAGCGCTGATGAACCTGAATCAGTC-3′
BgaA-Flanking	F	5′-GGGGATCTAAATTCTTCATCGGTT-3′
	R	5′AACCGATGAAGAATTTAGATCCCC3′
BgaA-T	F	5′-GCATACGTCAGGGATCCATGGGAAAGGCCATTGGAATCGG-3′
	R	5′-ATCAATCTCTAGATTAGTGATGGTGATGGTGATGACCTGTATTTGGTAAAGGCTTGCTCACATCTACTAA-3′
BgaA-T^{K96R}	F	5′-GCATCTGAGAGGAAAGAAGATGAAGCCGTAACTCCAAAAG-3′
	R	5′-CTTCATCTTCTTTCCTCTCAGATGCAATAGCCTCAGTTG-3′
BgaA-T^{K97R}	F	5′-TTGCATCTGAGAAGAGAGAAGATGAAGCCGTAACTCCAAAAG-3′
	R	5′-GCTTCATCTTCTCTCTTCTCAGATGCAATAGCCTCAGTTG-3′
BgaA-$T^{K96,97R}$	F	5′-CTATTGCATCTGAGAGGAGAGAAGATGAAGCCGTAACTCC-3′
	R	5′-TTCATCTTCTCTCCTCTCAGATGCAATAGCCTCAGTTGAAC-3′
BgaA-$T^{\Delta 102\text{-}109}$	F	5′-AAAGTGTCTGCTAAACCGGAAG-3′
	R	5′-GGCTTCATCTTCTTTCTTCTCAGA-3′
BgaA-T^{T103A}	F	5′-CGTAGCTCCAAAAGAGGAAAAAGTGTCTGCT-3′
	R	5′-ACTTTTTCCTCTTTTGGAGCTACGGCTTCATCTTCTTT-3′
BgaA-T^{P104Q}	F	5′-GAAGAAAGAAGATGAAGCCGTAACTCAAAAAGAGGAAAAAGTGTCTGCTAAAC-3′
	R	5′-TTCCTCTTTTTGAGTTACGGCTTCATCTTCTTTCTTCTCAGATGCAATAGCCT-3′
BgaA-T-GFP-TRE	F	5′-CGCGGCCACGCGTGGATCTTAGGCGTAGTCGGGCACG-3′
	R	5′-GGCTAGCTAGACAGGATCGCCACCATGGTGAGCAAGG-3′
$HysA^{Degron\text{-}BgaA}$	F	5′-GTAACTCCAAAAGAGGAAGCTCTAGGTGGAAACTTAGTTGAT-3′
	R	5′-GGCTTCATCTTCCTTGAATGGGTTATCAGTCGTCTTTCG-3′
PspA-Upstream	F	5′-CGTTAGATATCACAAGTTGTTGCATCG-3′
	R	5′-GGAGCCCATATAGTCATTTTCAG-3′
PspA-Downstream	F	5′-ATTAGGATCCGCCGATTAAATTAAAGCATG-3′
	R	5′-TTTTAGAGCTCGATTGAAGGTCGCTTGA-3′
PspA	F	5′-TCTCGGCTGCCGCTACGGATCCATGAATAAGAAAAAAATGATTTTAACAAGT-3′
	R	5′-ATCTCTTCTAGACTAGTGGTGATGGTGATGATGAACCCATTCACCATTGGCAT-3′
$PspA^{K314R}$	F	5′-AAGCTGACCTTAGGAAAGCAGTTAATGAGCCAGAAAAACC-3′
	R	5′-CATTAACTGCTTTCCTAAGGTCAGCTTCAGTTTTTTCTAA-3′
$PspA^{K315R}$	F	5′-AAGCTGACCTTAAGAGAGCAGTTAATGAGCCAGAAAAACC-3′
	R	5′-GCTCATTAACTGCTCTCTTAAGGTCAGCTTCAGTTTTTC-3′
$PspA^{K314R,K315R}$	F	5′-CTGAAGCTGACCTTAGGAGAGCAGTTAATGAGCCAGAAAA-3′
	R	5′-CATTAACTGCTCTCCTAAGGTCAGCTTCAGTTTTTTCTAATTC-3′

PspA$^{\Delta Degron}$	F	5′-GCACCAGCTGAACAACCAAAACCAGCGCCGG-3′
	R	5′-AGCTGGAGCTGGTTTTTCTGGCTCATTAACTGCTTTCT-3′
PspA^{T329A}	F	5′-CTCCAGAAGCTCCAGCCCCAGAAGCACCAGC-3′
	R	5′-GGGGCTGGAGCTTCTGGAGCTGGAGCTGGTTTTTCTGGCTCATTAAC-3′
HysA	F	5′-CTATATGGATCCATGGACTACAAGGACGACGACGATAAGCAAACAAAA ACAAAGAAGCT-3′
	R	5′-ATTTAATTAGCGGCCGCCTAGTGGTGATGGTGATGATGGTTGTTCTTTCC TCTACG-3′
HysA$^{Degron\text{-}PspA}$	F	5′-CCAGAAACTCCAGCCCCAGAATTCAAGGCTCTAGGTGGAAACTTAGTTG ATATGG-3′
	R	5′-TTCTGGAGCTGGAGCTGGTTTTTCTGGGTTATCAGTCGTCTTTCGGAAAT GTTCGGG-3′
HysA-upstream	F	5′-TCTAGATCTGCTTCCTTACCGTTGAC-3′
	R	5′-GGATCCTAGGAACTAAATCTCAAATTA-3′
HysA-downstream	F	5′-GGATCCTTTGTTCATCATCTAGATGA-3′
	R	5′-CTCGAGTTTCGCCTAAACTACTTCTAT-3′
pET28A- BgaA-T	F	5′-TCGATCGTCTAGATTTGTTTAACTTTAAGAAGGAGATATACATGG GGAAAGGCCATTGGAATCG-3′
	R	5′-TATCATTGCGGCCGCGGCGTAGTCGGGCACGTCGTAG GGGTAAAACAGTCTTTTCTTGTCCTT-3′
PspA- Flanking	F	5′-TGTTGCATCGTAGCTAAGGATTTAT-3′
	R	5′-CCCATCTATTCGTTTATTCAC-3′
PspA-Seq	F	5′-TGCAAAACTTGAAGATCAA-3′
HysA-Seq	F	5′-CAAGTGACCAATCCTTCTTCTCGTTA-3′
Chloramphenicol Resistance Cassette	F	5′-AGCCTTGGATCCATGCGTGAGAATGTTACAGT-3′
	R	5′-ATCCGGATCCTACAGTCGGCATTATCTCATA-3′
Spectinomycin Resistance Cassette	F	5′-ATCCGGATCCAATCTGATTACCAATTAGAATG-3′
	R	5′-CCGCGGATCCCATATATAATCTAGAATAAAATTAAC-3′
pGEX-4T-FBXW7	F	5′-TGGATCCCCGGAATTCATGAATCAGGAACTGCTCTCTG-3′
	R	5′- GTCGACCCGGGAATTCTCACTTCATGTCCACATCAAAG-3′
FBXW7A505C	F	5′- GCAGCAGTCTGCTGTGTTCAATATGATGGCAGGAGG-3′
	R	5′- TTGAACACAGCAGACTGCTGCAACATGACCCATCAA-3′
